# Frazzled/DCC Regulates Gap Junction Formation at a *Drosophila* Giant Synapse

**DOI:** 10.1523/ENEURO.0202-25.2025

**Published:** 2025-10-23

**Authors:** Juan Lopez, Jana Boerner, Kelli Robbins, Rodrigo F. O. Pena, Rodney Murphey

**Affiliations:** ^1^Department of Biological Sciences, Florida Atlantic University, Jupiter, Florida 33458; ^2^Stiles-Nicholson Brain Institute, Florida Atlantic University, Jupiter, Florida 33458

**Keywords:** computational, *Drosophila*, Frazzled/DCC, gap junction, Giant Fiber, synapse

## Abstract

Loss-of-function (LOF) Frazzled/DCC mutants disrupt synaptogenesis in the Giant Fiber (GF) System of *Drosophila*. We observed weaker physiology in LOF male and female specimens, characterized by longer latencies and reduced response frequencies between the GFs and the motor neurons. These physiological phenotypes are linked to a loss of gap junctions in the GFs, specifically the loss of the shaking-B(neural+16) isoform of innexin in the presynaptic terminal. We present evidence of Frazzled's role in gap junction regulation by utilizing the UAS-GAL4 system in *Drosophila* to rescue mutant phenotypes. Expression of various UAS-Frazzled constructs in a Frazzled LOF background was used to dissect the role of different parts of the Frazzled receptor in the assembly of electrical synapses. Expressing Frazzled’s intracellular domain in Frazzled LOF mutants rescued axon pathfinding and synaptogenesis. This is supported by the complementary result that Frazzled fails to rescue synaptic function when the transcriptional activation domain is disrupted, as shown by the deletion of the highly conserved intracellular P3 domain or by a construct with a point mutation in the highly conserved P3 domain known to be required for transcriptional activation. A computational model clarifies the role of gap junctions and the function of the GF System. The present work shows how various domains of a guidance molecule regulate synaptogenesis through the regulation of synaptic components.

## Significance Statement

Loss-of-function (LOF) Frazzled/DCC mutants demonstrate that the gene regulates synaptogenesis in the Giant Fiber (GF) System of *Drosophila*. In *frazzled* LOF mutants, we observe weaker physiology, characterized by longer latencies and reduced response frequencies between the GFs and the motor neurons. These physiological phenotypes are linked to a loss of gap junctions in the GFs. A GF computational model is provided to test the role of gap junctions and the function of the GF System. We present evidence of Frazzled's role in gap junction regulation by utilizing the UAS-GAL4 system in *Drosophila* to rescue mutant phenotypes. We also show that this effect can be computationally modeled, supporting our findings and presenting a novel role of Frazzled.

## Introduction

Various ligand–receptor pairs are involved in axon guidance and pathfinding, and recently, some have been shown to have a second function during synaptogenesis. Netrin is a well characterized and conserved axon guidance cue that was the first of its kind to be discovered, and its receptor is broadly known as Frazzled/DCC (Hedgecock et al., 1990; Serafini et al., 1994; Mitchell et al., 1996; Phelan et al., 1998; Phelan and Starich, 2001; Boyer and Gupton, 2018). *Drosophila* Frazzled and its human homologs, DCC and neogenin (Kolodziej et al., 1996), belong to the immunoglobulin (Ig) superfamily of transmembrane-associated proteins defined by the Ig domain consisting of ∼100 amino acids arranged in the extracellular space and an intracellular domain (ICD) that can serve as a transcriptional activator (Faissner, 2009). The Netrin–Frazzled pair is involved in synapse formation in worms (Colon-Ramos et al., 2007) and flies (Orr et al., 2014).

The Giant Fiber (GF) System of *Drosophila* is one of the animal kingdom's most extensively studied synaptic circuits (Power, 1948; Thomas and Wyman, 1983; Blagburn et al., 1999; Allen et al., 2006). Each GF has a cell body and dendrites in the brain and extends a large axon out of the brain along the midline to the mesothoracic neuromere (Allen et al., 1998). There it forms a mixed electrical–chemical synapse to the tergotrochanteral motoneurons (TTMn), resulting in a reliable, high-speed circuit that signals to the jump muscles, establishing the rapid escape behavior of flies (Thomas and Wyman, 1983; Blagburn et al., 1999; Allen et al., 2006; Phelan et al., 2008; Von Reyn et al., 2014). The mixed synapse is comprised of a chemical component that releases acetylcholine (Allen and Murphey, 2007) and an electrical component mediated by innexins produced from the gene *shaking-B* (Mark and King, 1983; Thomas and Wyman, 1983; Phelan et al., 1996, 2008; Jacobs et al., 2000). In the fly, alternative splicing of *shaking*-*B* produces three different innexin proteins: shaking-B(lethal), shaking-B(neural), and shaking-B(neural+16) (Allen et al., 2006). Like the vertebrate connexins, the innexin gap junctions are composed of six protein subunits in the presynaptic and six in the postsynaptic membranes. The shaking-B(neural+16) innexin is found in the presynaptic GFs, while the shaking-B(lethal) isoform is thought to be expressed in the postsynaptic TTMn, and together these heterotypic channels form rectifying junctions (Phelan et al., 2008).

### Axon guidance molecules and synapse assembly

The Netrin receptor Frazzled and its vertebrate homologs DCC and neogenin are known to play roles beyond axon guidance, including synaptogenesis, regeneration, and cell migration (Colon-Ramos et al., 2007; Flores, 2011; Ylivinkka et al., 2016). Previous work in our lab primarily focused on Netrin showed that Netrin and Frazzled regulate gap junctions in the GFs (Orr et al., 2014). The receptor Frazzled canonically binds Netrin on growth cones via its extracellular domain and serves as an attractant (Kolodziej et al., 1996). Recently, a novel, noncanonical, Netrin-independent role of Frazzled was found, where the ICD of Frazzled was shown to function as a transcriptional activator of the gene *commissureless* in embryonic *Drosophila* (Neuhaus-Follini and Bashaw, 2015; Zang et al., 2022). Further exploration of Frazzled’s ICD revealed that the P3 domain was responsible for this transcriptional effect (Neuhaus-Follini and Bashaw, 2015). We hypothesize that a similar effect occurs in the GFs, where Frazzled may be regulating GJs in the GFs. Here we present evidence for the role of Frazzled in synapse formation, structure, and function. Using UAS-GAL4–driven Frazzled constructs, we dissect the molecular requirements of Frazzled for gap junction assembly in the CNS of adult *Drosophila*. We demonstrate that Frazzled’s ICD is necessary and sufficient to produce shaking-B gap junctions in the GFs by quantifying changes in physiology and gap junction production across our various rescue experiments.

## Materials and Methods

### Fly stocks

**Figure 1.**
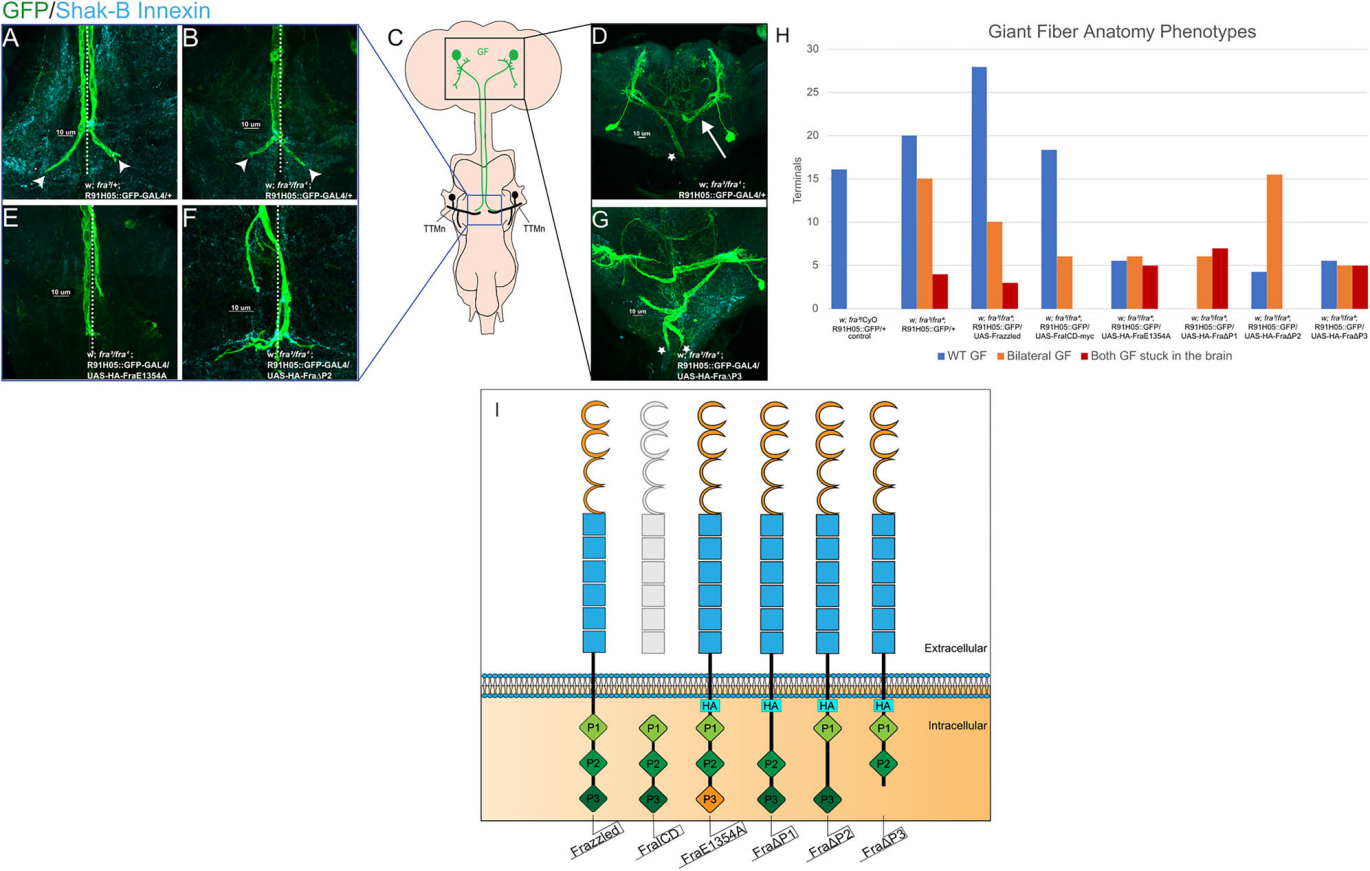
Axon guidance phenotypes and construct schematics. ***A–G***, Confocal images of the various axon guidance phenotypes found in *frazzled* LOF specimens. Midlines are shown in dotted lines. ***A***, Wild-type-appearing GFs and terminals (arrowheads). ***B***, A single left GF forms a bilateral terminal (arrowheads). ***C***, Schematic of the GF system. The GF somata are labeled green, and the axons extend along the midline to form terminals that synapse onto ipsilateral TTMn partners in black. ***D***, The missing left GF from B can be seen in the brain (arrow), but its axon does not leave the brain. The right GF extends an axon seen in ***B***. ***E***, Rarely occurring GFs that do not form traditional bends toward their TTMn partners. ***F***, Disrupted axons in GFs driving UAS-HA-FraΔP2. ***G***, Neither GF grows out of the brain. ***H***, The frequency of different axon guidance phenotypes for each genotype tested. ***I***, Diagram showing the structural differences between the UAS constructs used in our experiments. The extracellular domain for each construct contains four Ig C2 repeats (orange) and six fibronectin III repeats (blue), while the ICD consists of the conserved P1, P2, and P3 domains (green). UAS-FraE1354A contains an HA tag that does not influence synaptic function (seen in Extended Data Fig. 1-1).

*Drosophila melanogaster* were raised at 25°C, and data were collected from both male and female flies. Adults 2–4 d old were selected for testing. We generated a *frazzled* loss-of-function (LOF) mutant using *fra^3^* and *fra*^4^ null alleles (Kolodziej et al., 1996; Zang et al., 2022). When either allele is homozygous, the mutant is lethal; however, the *trans*-heterozygous mutant (*fra*^3^/*fra*^4^) has a low survival rate, and we used these escapers for experiments. The UAS-GAL4 system was used to fluorescently label the GFs using the R91H05::GFP-GAL4 as our GF-GAL4 driver (Fig. 1*A,C*; Jenett et al., 2012). The tested fly lines are listed in Table 1. The following parent lines were also generated and tested (data not shown): w; *fra^3^/*CyO; R91H05::GFP-GAL4/+, w; *fra^3^/*CyO; R91H05::GFP-GAL4/Tb, w; *fra^4^*/CyO; R91H05::GFP-GAL4/Tb, and w; *fra^4^*/Gla, Bc; R91H05::GFP-GAL4/TM3. The following published transgenic lines were used in this study: UAS-FraICD-myc, *fra*^3^/CyObb, UAS-Frazzled-Myc/TM2, UAS-HA-FraE1354A, UAS-HA-FraΔP1, UAS-HA-FraΔP2, and UAS-HA-FraΔP3 (Fig. 1*I*; Garbe and Bashaw, 2004; Neuhaus-Follini and Bashaw, 2015).

**Table 1.** A KS2D2S test to identify differences between *frazzled* LOF mutants and LOF flies driving different UAS-Frazzled rescue constructs

KS2D2S					
	Sample 1 (*n* = 35 terminals)	Sample 2	Sample 2 Terminals	*p*-value (Response Latency versus GJ%)	*p*-value (Response Frequency versus GJ%)
1	*frazzled* LOF mutant (w; *fra*^3^/*fra*^4^; R91H05::GFP/+)	*frazzled* heterozygous sibling (w; *fra*^3^/CyO; R91H05::GFP/+)	*n* = 16	0.0001*	0.0001*
2	*frazzled* LOF mutant (w; *fra*^3^/*fra*^4^; R91H05::GFP/+)	Full-length Frazzled (w; *fra*^3^/*fra*^4^; R91H05::GFP/UAS-Frazzled)	*n* = 38	0.0373*	0.0076*
3	*frazzled* LOF mutant (w; *fra*^3^/*fra*^4^; R91H05::GFP/+)	Frazzled’s intracellular domain (w; *fra*^3^/*fra*^4^; R91H05::GFP/UAS-FraICD)	*n* = 23	0.0007*	0.0004*
4	*frazzled* LOF mutant (w; *fra*^3^/*fra*^4^; R91H05::GFP/+)	Point-mutated Frazzled (w; *fra*^3^/*fra*^4^; R91H05::GFP/UAS-HA-FraE1354A)	*n* = 13	0.0628	0.0404*
5	*frazzled* LOF mutant (w; *fra*^3^/*fra*^4^; R91H05::GFP/+)	Frazzled P1 domain deletion (w; *fra*^3^/*fra*^4^; R91H05::GFP/UAS-HA-FraΔP1)	*n* = 7	0.0013*	0.0153*
6	*frazzled* LOF mutant (w; *fra*^3^/*fra*^4^; R91H05::GFP/+)	Frazzled P2 domain deletion (w; *fra*^3^/*fra*^4^; R91H05::GFP/UAS-HA-FraΔP2)	*n* = 20	0.0002*	0.0054*
7	*frazzled* LOF mutant (w; *fra*^3^/*fra*^4^; R91H05::GFP/+)	Frazzled P3 domain deletion (w; *fra*^3^/*fra*^4^; R91H05::GFP/UAS-HA-FraΔP3)	*n* = 12	0.3085	0.5607
8	*frazzled* LOF mutant (w; *fra*^3^/*fra*^4^; R91H05::GFP/+)	Wild-type unablated (w; + ; R91H05::GFP/10×-UAS-GFP)	*n* = 8	0.0055*	0.0098*
9	*frazzled* LOF mutant (w; *fra*^3^/*fra*^4^; R91H05::GFP/+)	Wild-type ablated (w; + ; R91H05::GFP/10×-UAS-GFP)	*n* = 22	0.0282*	0.0009*
10	*frazzled* LOF mutant (w; *fra*^3^/*fra*^4^; R91H05::GFP/+)	*shak-B*^2^ heterozygous sibling ablation (*shak-B*^2^/Fm6; + ; R91H05::GFP/R91H05::GFP)	*n* = 14	0.3688*	0.0076*
11	*frazzled* LOF mutant (w; *fra*^3^/*fra*^4^; R91H05::GFP/+)	*shak-B*^2^ ablation (*shak-B*^2^/*shak-B*^2^; + ; R91H05::GFP/R91H05::GFP)	*n* = 10	0.0019*	0.0003*

The individual, independent comparisons made with the KS2D2S test revealed whether there are differences in the distributions of response latency averages and the proportion of volume taken up by gap junction antibody in GF terminals, with significant differences shown by asterisks (*). The KS2D2S comparing response frequencies to the proportion of volume taken up by gap junction antibody in GF terminals shows the same significant differences as the latency test, except for UAS-FraE1354A (see results).

Crosses can generate up to eight different genetic siblings. In each cross, two of these genetic siblings are the *trans*-heterozygous *frazzled* LOF mutants. Heterozygous *frazzled* LOF control siblings also appeared as two genetic siblings and were phenotypically wild type, and we used the w; *fra*^3^/Gla, Bc; R91H05::GFP-GAL4/Tb sibling as our sibling control. This sibling is phenotypically wild type and appears in all the crosses generated. Since the data collected from this sibling is consistent, we grouped the data collected across different crosses together for easier comparisons.

### Electrophysiology

**Figure 2.**
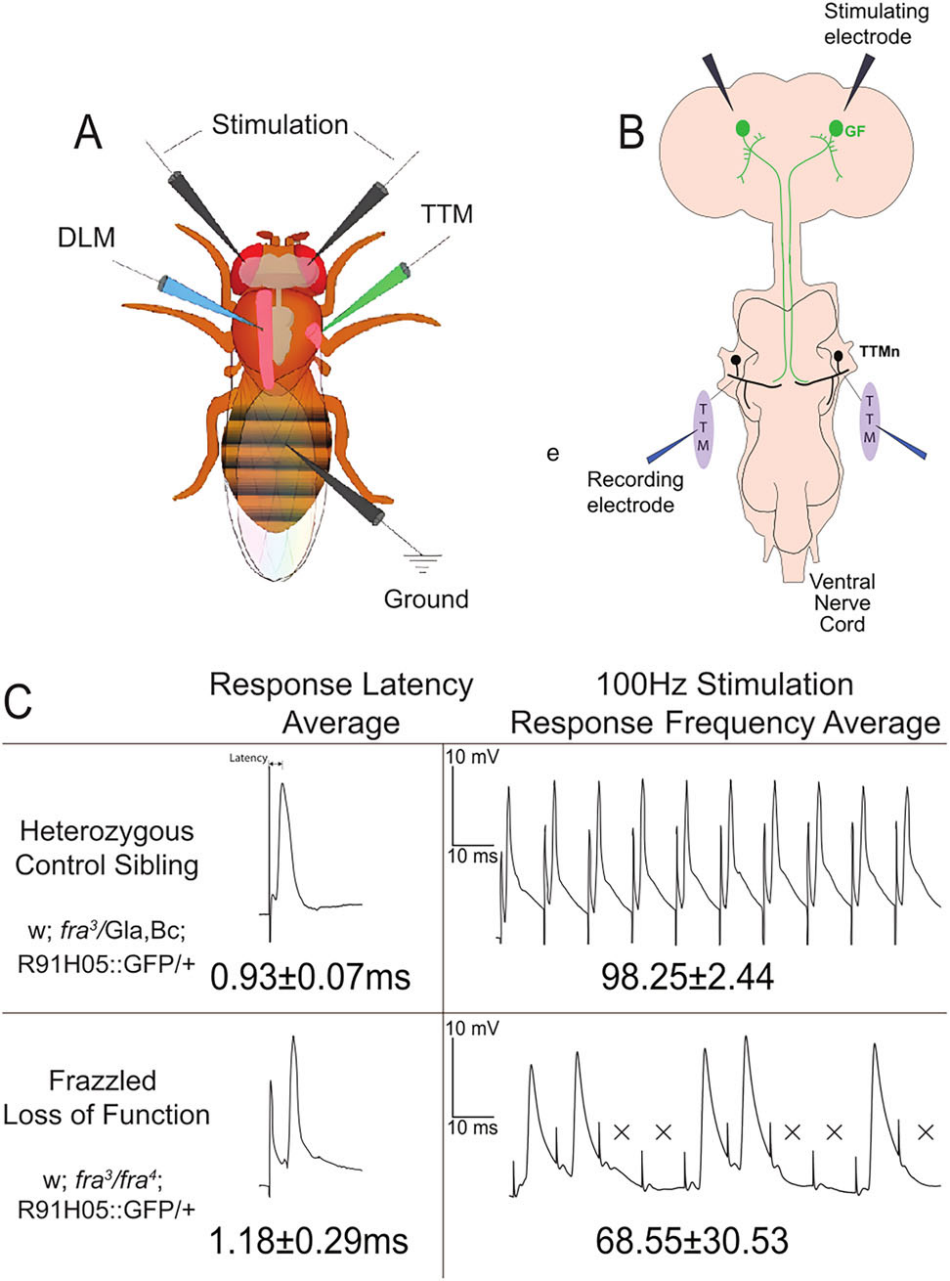
Recording from the GF System. ***A***, The GF System (GFS) relays signals from the brain to the jump muscles in the thorax. We insert stimulating electrodes through the eyes to extracellularly stimulate the GF and insert recording electrodes in the jump muscle to record latency and response frequency for the circuit (see Materials and Methods). ***B***, Schematic of the GF system, with stimulating electrodes in the brain and recording electrodes placed extracellularly in the TTM. ***C***, Latencies are recorded 10 times with 1 s intervals between stimuli. Wild type is defined by latency averages under 1.00 ms. When we stimulate the GFs 10 times in 10 s at 100 Hz, wild-type samples have a consistent response frequency averaging over 90%. The top panels are from heterozygous *frazzled* control sibling samples and are wild type. In contrast, the bottom panels are *frazzled* LOF mutants, which perform significantly worse in comparison (missing responses indicated by the X).

Flies were embedded ventral side down in dental wax after anesthetizing with CO_2_. Physiological recordings were obtained bilaterally using glass microelectrodes placed directly into the tergotrochanteral jump muscle (TTM) and dorsal longitudinal muscle (Fig. 2*A*,*B*; Allen et al., 2006; Allen and Godenschwege, 2010; Orr et al., 2014; Augustin et al., 2017). Each microelectrode was filled with O'Dowd's *Drosophila* saline (Gu and O’Dowd, 2006). GFs were stimulated extracellularly using Grass stimulators with tungsten wire electrodes placed in the brain through the eyes, and a tungsten wire ground electrode was placed in the fly's abdomen (Fig. 2*A*,*B*; Allen and Godenschwege, 2010). An Axon Digidata 1440A Data Acquisition System was used to digitize the data, and recordings were collected using the Clampex software (Molecular Devices). Muscle response latency was reported in milliseconds, and typical wild-type TTM latencies are <1.00 ms. Response frequencies were obtained by initiating a train of 10 stimuli at 100 Hz every second for 10 trains. These results were reported as an average percentage of successful muscle responses per stimuli for each animal. Wild-type response frequencies are defined as ≥90% responses to train stimulation at 100 Hz.

Recorded responses to stimuli result from an individual GF terminal-to-TTMn connection. In *frazzled* mutant animals, approximately half of the GF axons grow aberrantly in the brain and never reach the ventral nerve cord. The remaining GF grows to the VNC and forms a bilaterally symmetric terminal. The recordings are taken from the TTM, and each response is assigned to a terminal (left or right), and each terminal is treated as a separate unit for the purposes of statistics throughout the results, whether it belongs to a bilateral GF or a wild-type–appearing GF. The physiology and anatomy were assessed separately for each terminal unit.

Principal component analysis (PCA) was employed to reduce dimensionality and extract the underlying shared variance of the three measures. In our analysis, PCA was performed on the standardized values (*z*-scores) of Latency Average, Gap Junction %, and 100 Hz Average. The global PCA yielded three components with explained variance ratios of ∼54.0, 28.6, and 17.4% for PC1, PC2, and PC3, respectively. In addition, a permutation test (1,000 iterations) was conducted to assess the significance for each component. The test confirmed that PC1 was statistically greater than what would be expected by chance (*p* < 0.001).

Although PCA was initially applied to the entire filtered dataset to assess overall dimensionality, subsequent analyses focused on genotype-specific comparisons due to limitations in the sample size and group balance. In many genotypes, one of the groups (wild type or bilateral) was underrepresented or absent. Consequently, robust statistical comparisons between wild-type and bilateral groups were restricted to the genotype w; *fra*^3^/*fra*^4^; R91H05::GFP/+, which had sufficient representation in both subgroups. We extracted the PCA scores for each individual unit terminal and conducted MANOVA of the PCA scores (PC1, PC2, and PC3) with “wild type or bilateral” as the independent factor. MANOVA yielded *F* = 0.261 (*p* = 0.853), indicating no significant difference between the groups. Consequently, each principal component was then compared between wild-type and bilateral individuals using *t* tests. The results were nonsignificant for PC1 (*t* = −0.784; *p* = 0.439), PC2 (*t* = −0.041; *p* = 0.968), and PC3 (*t* = −0.438; *p* = 0.664), suggesting that, in the composite PCA space, the two groups do not differ meaningfully. After adjusting the three univariate PC tests within this genotype for multiple comparisons (Bonferroni, Holm, and Benjamini–Hochberg), none of the PC comparisons remained significant (adjusted *p* ≥ 0.968), consistent with the nonsignificant MANOVA.

### Immunohistochemistry

Flies were removed from dental wax after electrophysiology tests were concluded and dissected in phosphate-buffered saline (PBS). We opened the thorax and abdomen of the flies so that the ventral nervous system was exposed (Boerner and Duch, 2010; Boerner and Godenschwege, 2010, 2011), and the head case was also opened to expose the brain. Samples were fixed in 4% paraformaldehyde for 45 min, washed in PBS overnight and then washed in a PBS 0.5% Triton X-100 for 2 h at room temperature. The GFs were labeled using primary antibodies: rabbit anti-HA antibody (1:500; Covance; Extended Data Fig. 1-1), mouse anti-GFP (1:500; Invitrogen), and rabbit anti-shaking-B (1:500; Biomatik). Our anti-shaking-B was generated by Biomatik following a published peptide sequence (Phelan et al., 1996) and binds to a consensus sequence shared by all shaking-B isoforms. The sequence of the peptide, including the added cysteine (underlined) was CQHHRVPGLKGEIQD (amino acids 347–360 and 358–371) in the sequence of the neural (Krishnan et al., 1993) and lethal (Crompton et al., 1995) forms of the protein, respectively. Primary antibodies were prepared using PBS with Triton X-100 0.3% and bovine serum albumin 3%. After incubating for two nights at 4°C, samples were washed for 2 h in PBS, and secondary antibodies with conjugated fluorophores were applied overnight to detect proteins under confocal microscopy: goat anti-rabbit Cy5 (1:500) and goat anti-mouse Alexa Fluor 488 (1:500) at 4°C. Each sample was then washed in PBS for 2 h and dehydrated in an ethanol series for 10 min each in 50, 70, 90, and 100% ethanol and mounted on glass slides filled with methylsalycylate and sealed with a coverslip for imaging.

### Imaging

Samples were imaged on a Nikon A1R laser scanning confocal microscope. Samples were magnified using a 60× oil immersion objective and scanned with *z*-step sizes of 0.125 µm. Laser channels used were 488 and 640 nm. Channels were imaged in series using filters to avoid cross-excitation of close wavelengths with 2× integration. Maximum intensity *z*-stack projection images were created in the Nikon NIS Elements software; contrast and brightness were adjusted to increase image clarity.

**Figure 3.**
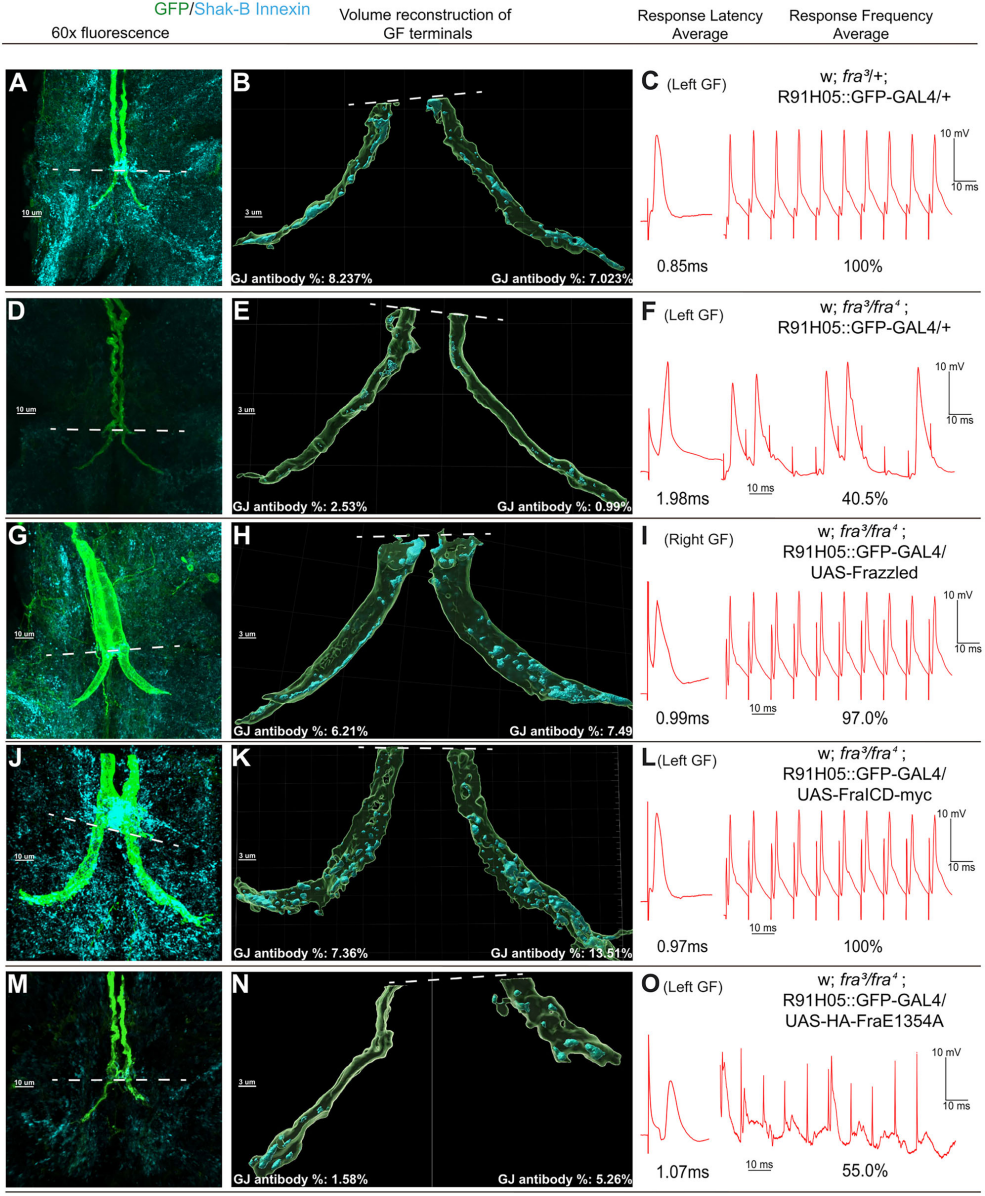
Structure of GF axons when driving different UAS constructs in a *frazzled* LOF background. Each row has images from an individual sample for a given genotype. A representation of each Frazzled construct is seen in Figure 1*I*. First column (***A, D, G, J***): Compressed *z*-stack maximum projection image of the GF terminals labeled with GFP and shaking-B(neural+16) gap junctions labeled with shaking-B antibody, which binds to all shaking-B isoforms. The dotted line marks the PSI region and the anterior limit of the unit terminals. Second column (***B, E, H, K***): Volume reconstructions of the corresponding GF terminals posterior to the PSI region. The reconstruction generates volumes for the GF terminals and the gap junction antibodies within the terminals, which are included for each sample. Third column (***C, F, I, L, O***): Genotype of the samples in each row and that sample’s latency and response frequency average.

To analyze the structure and content of the GFs, *z*-stack files were imported into the IMARIS (v10.2.0) software. Before rendering, background subtraction was applied to both laser channels [640 nm for green fluorescent protein (GFP) and 488 nm for shaking-B]. Channels were surfaced using a dynamic thresholding method within IMARIS that matched fluorescence in each *z*-dimension on a sample-by-sample basis. Masks of each channel were created for each surface to detect colocalization of GFP and shaking-B in the terminals. Terminals were defined as the area of the GFs caudal to the peripherally synapsing interneuron (PSI) region (Fig. 3, dotted lines). Images were acquired in volume view and exported in TIFF format.

### Statistical analysis

All statistical analyses were conducted in Python using packages including pandas, NumPy, scikit-learn for PCA and data scaling, statsmodels for MANOVA, and SciPy for *t* tests and Hotelling's T^2^ calculations. A 2D two-sample Kolmogorov–Smirnov (KS2D2S) test was used to examine the differences between genotypes using latency and the percentage of the synaptic terminal filled with antibody to the gap junction. The KS2D2S test was performed using Python's NumPy and SciPy libraries and carried out using the scipy.stats library in Python, with a significance level (alpha) set at 0.05. Our hypothesis states that Frazzled has a role in assembling the gap junction component of the GFs, and we show this by comparing response latencies, response frequencies, and the proportion of terminal volume occupied by gap junction antibody in various genotypes.

### Cell ablation

In some *frazzled* mutants, only one GF grows from the brain to the thorax, and the GF usually splits to form a bilateral terminal. To investigate the bilateral terminal in a wild-type background, we ablated one GF and characterized the structure and function of the remaining GF. Late third instar larvae were positioned dorsal side up on a multiphoton microscope stage, and the GF cell bodies were identified in the brain for GF ablation. After successfully laser ablating the cell (focusing laser light onto the cell until it is visibly ruptured), the larvae were placed in a food vial and raised under standard conditions until they eclose from the pupal case [see Boerner and Godenschwege (2011) for details]. Adults were recovered after ablation, and the strength of the bilateral connections was assessed as described above. After physiological recordings were performed, specimens were dissected to expose the CNS, and GFs were dye-injected with a mix of TRITC-Dextran (Thermo Fisher Scientific) and NEUROBIOTIN (Vector Laboratories). The large Dextran molecule labels the GF axon and terminal but does not pass through gap junctions. NEUROBIOTIN can cross gap junctions between GF and TTMn; NEUROBIOTIN was detected postfixation using streptavidin conjugated to a fluorophore (streptavidin–Alexa Fluor 647, 1:500; Thermo Fisher Scientific) following standard permeabilization and blocking (0.1% Triton X-100, 5% normal goat serum). This NEUROBIOTIN dye-coupling confirmed intact gap junctions between the two neurons. Finally, the percentage of the terminal occupied by antibodies to the gap junction was measured and compared with other treatments.

### Computational modeling of the GF

Our study utilizes a computational model composed of four interconnected neuronal compartments designed to investigate the effects of synaptic strength, gap junction conductance, and external stimuli on neuronal firing behaviors (Follmann et al., 2015; Augustin et al., 2019). The first compartment receives an excitatory stimulus, *I*_stim_, designed as a pulse current (10 Hz) combined with a zero-mean and unit-variance random Gaussian noise to mimic the variability observed in biological neuronal inputs. This external stimulation propagates through the other compartments (axial resistance) and can trigger action potentials in the postsynaptic neuron compartment, depending on the strength of the gap junction or chemical synapse that connects these neurons or the frequency and amplitude of the input signal.

### Neuronal dynamics and gating variables

The model is based on Hodgkin–Huxley formalism, detailed through dynamic variables: membrane potential (*V*) and gating variables (*n*, *m*, and *h*). Ionic currents and synaptic inputs influence each compartment’s membrane potential (*V*) (*I*_syn_), defined as follows:

$$C_{m}\frac{dV}{dt}=-(I_{Na}+I_{K}+I_{L}+I_{\mathrm{syn}})+I_{\mathrm{stim}},$$

where *C_m_* denotes the membrane capacitance and *I_Na_*, *I_K_*, and *I_L_* represent the sodium, potassium, and leak currents. *I_syn_* and *I_stim_* symbolize synaptic and external stimulation currents. The gating variables (*x* = *n,m,h*) are defined by the following:

$$\frac{dx}{dt}=\frac{x_{\inf}(V)-x}{\tau_{x}(V)},$$

where the steady-state values of the gating variables for a given membrane potential *V* is given as follows:

$$x_{\inf}(V)=\frac{1}{1+e^{(V_{1/2}-V)/k\;}},$$

with $V_{1/2}=-53$ Mv and k=15 for n, $V_{1/2}=-40$ Mv and k=15 for *m*, and $V_{1/2}=-62$ Mv and k=−7 for *h*. The corresponding voltage-dependent time constants are governed by $\tau_{n}=1.1+4.7\exp\;\left(-{\left(\frac{-79-V}{50}\right)}^{2}\right)$, $\tau_{m}=0.04+0.46\exp\;\left(-{\left(\frac{-38-V}{30}\right)}^{2}\right)$, and $\tau_{h}=1.2+7.4\exp\;\left(-{\left(\frac{-67-V}{20}\right)}^{2}\right)$. Reversal potentials are −85 mV for leak, −74 mV for potassium, and 65 Mv for sodium.

### Chemical and electrical signal modeling

The model uses chemical (*I*_chem_) and electrical (*I*_gap_) synapses to simulate the rapid escape response of *Drosophila* GFs. The chemical synaptic current, *I*_chem_, is defined as follows:

$$I_{\mathrm{chem}}\;=\;g_{\mathrm{syn}}\;\cdot \;s\;\cdot \;(V_{\mathrm{post}}\;-\;V_{\mathrm{syn}}),$$

where *g*_syn_ represents the maximum synaptic conductance, set to 0*.*1 in the model, *V*_post_ is the postsynaptic compartment’s membrane potential, and *V*_syn_ is the synaptic reversal potential, set to 0 mV, indicating an excitatory synapse. *s* represents the fraction of activated receptors or open synaptic channels at any given time. The dynamics of *s* is governed by the following equation:

$$\frac{ds}{dt}=\frac{1+\tanh\;(V_{\mathrm{pre}}/4)}{2}\cdot \frac{1-s}{\tau_{\mathrm{rise}}}-\frac{s}{\tau_{\mathrm{decay}}},$$

where *V*_pre_ is the membrane potential of the presynaptic compartment and *τ*_rise_ and *τ*_decay_ are the time constants for the synaptic activation and deactivation, respectively. In this model, *τ*_rise_ = 0*.*1 ms and *τ*_decay_ = 3 ms are used to simulate the synaptic response kinetics. For *I*_gap_, we assume *s* = 1, and the electrical synaptic conductance value (*g*_gap_) is obtained from the percentage of terminal volume filled (*p*), allowing for a coupling strength that connects with the experimental samples through a sigmoid relationship:

$$g_{\mathrm{gap}}=\frac{L_{\min}+(L_{\max}-L_{\min})}{1+\exp\;(-k\cdot (\;p-x_{0}))}.$$

Here, *L*_max_ and *L*_min_ represent biologically grounded maximum and minimum gap junction conductance values, respectively. For values of *p* > 10%, we assume *g*_gap_ = *L*_max_. The parameter *k* controls the steepness of the sigmoidal function, and *x*_0_ determines its midpoint. This formulation ensures that the conductance transitions smoothly from *L*_min_ to *L*_max_ as the percentage of terminal volume filled increases, capturing the biological variability in electrical coupling.

### Code accessibility and implementation

The code/software described is available freely online at GitHub through the following link: https://github.com/Saint-Sam/Drosophila-Giant-Fiber-Compartment-Model. The code is also provided as extended data supporting Figure 5 as Extended Data Figure 5-1. All simulations were implemented using the Python (3.12) programming language in a Jupyter Labs environment (v7.3.2) on a Mac desktop computer (MacOS Sonoma version 14.6.1), with Euler integration method for the differential equations with a time step of 1 ms for single, latency (10 ms) simulations and 100 ms for 100 Hz simulations (Fig. 5).

10.1523/ENEURO.0202-25.2025.d1 Data 1 **Computational Model of the GFS.** This Python (v3.12) code models the Giant Fiber system and the variable responses generated when changes to gap junction signaling are made. The code was developed in a Jupyter Notebooks environment (v7.3.2). Download Data 1, ZIP file.

## Results

### Axon pathfinding is disrupted in *frazzled* LOF mutants

In adult *Drosophila*, GF axons grow posteriorly from the brain through the connective and along the midline to the ventral nerve cord to form an ipsilateral synaptic terminal with their TTMn partners in the second thoracic segment (Fig. 1*C*). Previous studies in our lab found that GF axon pathfinding and synaptogenesis were disrupted in *netrin* LOF and *frazzled* LOF mutant flies (Orr et al., 2014). To examine the role of Frazzled in synaptogenesis more closely, we revisited this analysis and analyzed the GF system in *frazzled* mutants. To provide mechanistic insight into how specific domains of Frazzled regulate gap junction assembly, we expressed UAS-Frazzled constructs (illustrated in Fig. 1*I*) in mutant backgrounds and assessed their ability to rescue the mutant phenotypes. In Figure 1*A–G*, we present the range of GF anatomical phenotypes seen in *frazzled* LOF flies (*fra*^3^/*fra*^4^) and quantify the distribution of phenotypes in Figure 1*H*. In approximately half of the LOF flies, the GFs appear wild type, and each axon innervates the ipsilateral TTMn (*n* = 16 terminals; Fig. 1*A*,*H*). In the other half of the mutant flies, we find one phenotype never seen in wild-type animals, where one GF extends an axon to the thorax but the other never leaves the brain (*n* = 15 terminals; Fig. 1*D*). The GF that grew to the thorax formed a bilateral terminal to innervate both TTMn targets (Fig. 1*B*,*F*; Boerner and Godenschwege, 2011; Orr et al., 2014; Kennedy and Broadie, 2018). The bilateral terminal is a direct result of one GF being lost in the brain and an indirect result of the guidance errors caused by the *frazzled* LOF mutation. Finally, the most severe pathfinding phenotype seen in a few LOF mutants occurred when both GFs grow long, twisted axons that never leave the brain, which we called “stuck-in-the-brain” (*n* = 4 GFs; Fig. 1*G*). As a result, we record no motor responses to stimulation of these flies and cannot measure changes in gap junction antibody volume at the GF terminals. These flies are excluded from statistical analysis. The proportion of the various phenotypes for each genotype is recorded in Figure 1*H*.

10.1523/ENEURO.0202-25.2025.f1-1 Figure 1-1 **HA- tag immunolabeling in a *frazzled* LOF mutant driving expression of UAS-HA-FraE1354A in the Giant Fibers.** Figures show expression of UAS-HA-FraE1354A genetic construct. All panels are the same genotype. The top and bottom rows are different samples. **A)** Sample showing expression of GFP and anti-HA in the brain of *Drosophila*, with GFP in green and anti-HA in cyan. **B)** GFP expression channel. **C)** Anti-HA expression channel. **D)** Different sample showing expression of GFP and anti-HA in a single GF. **E)** GFP expression channel. **F)** Anti-HA expression channel. Download Figure 1-1, TIF file.

### LOF mutants exhibit defective physiology

**Figure 4.**
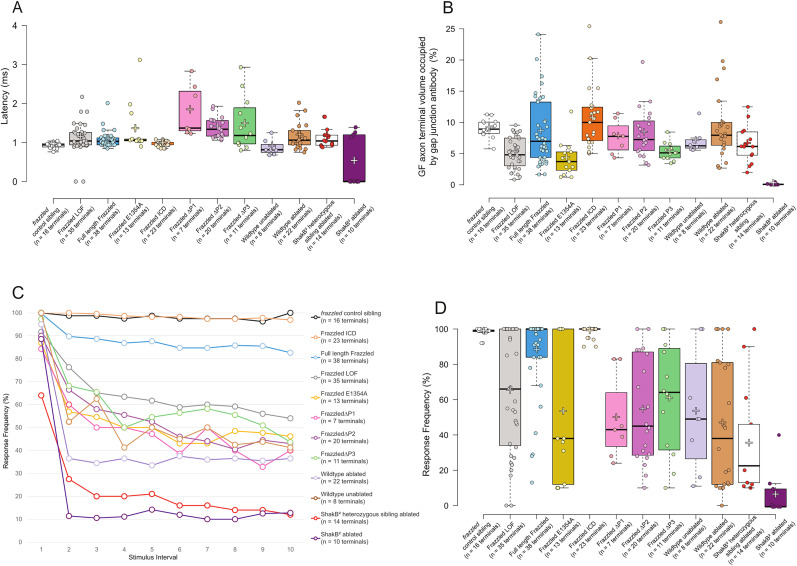
Comparison of latency, response frequency, and gap junction antibody proportion in GF terminals for all genotypes tested. ***A***, Latency comparison for all genotypes tested. Each point is the latency average of 10 trials from one side (unit terminal) of one specimen. ***B***, We measured GF terminal and gap junction antibody volume by generating a 3D rendering of *Z*-stack confocal images using the IMARIS software. We plotted the percent of a GF terminal occupied by gap junction antibody for each genotype tested. ***C***, Left- and right-side response frequency average for each fly of each genotype tested. ***D***, Response frequency comparison for all genotypes tested. Each point is the response average for a unit terminal stimulated 10 times at 100 Hz. Center lines (***A, B, D***) show the medians; box limits indicate the 25th and 75th percentiles as determined by the R software; whiskers extend 1.5 times the interquartile range from the 25th and 75th percentiles; crosses represent sample means; data points are plotted as open circles.

We next analyzed the physiology of the *frazzled* LOF flies and compared them with heterozygous sibling controls (w; *fra*^3^*/*Gla, Bc; R91H05::GFP-GAL4/Tb) that were used as the control in all the subsequent crosses we generated. When we apply a suprathreshold stimulus to the GFs of *frazzled* control siblings and record from the jump muscles, their response latencies average 0.93 ms (SD, 0.07; *n* = 16 terminals; Fig. 2*C*; Fig. 4*A*). We also stimulate the GFs 10 times in 100 ms at 100 Hz to determine the following frequency. Control siblings respond to each stimulus in 98.3% of the trials (SD, 2.44; *n* = 16 terminals; Fig. 2*C*; Fig. 4*D*). In contrast, *frazzled* LOF mutant flies exhibit longer response latencies than their control siblings (1.18 ms; SD, 0.30; *n* = 35 terminals; Fig. 2*C*; Fig. 4*A*) and reduced average following frequencies (68.55%; SD, 30.53; *n* = 35 terminals; Fig. 2*C*; Fig. 4*D*).

### Gap junctions are disrupted in *frazzled* LOF mutants

Previous experiments quantified the gap junction immunohistological label volume in the mutant and wild-type GFs (Orr et al., 2014). To revisit Orr’s results, we ordered a custom shaking-B innexin antibody and examined the distribution of gap junctions within the GFP-labeled terminals in mutant and control flies (see Fig. 3 and Materials and Methods). We measured the volume of gap junction staining within the IMARIS 3D synaptic terminal renderings. We defined the synaptic terminal as the region of the terminal posterior to the contact region (the inframedial bridge) for the GF and PSI (peripheral synapsing interneuron; Fig. 3, dashed white lines). The differences are visible in the volume renderings, such as Figure 3, *B* and *E*. In control siblings, gap junction antibody occupies 9.04% (SD, 1.33; *n* = 16 terminals; Fig. 4*B*) of GF terminal volume on average, and in the *frazzled* LOF mutants, gap junction antibody occupies 5.31% on average (SD, 2.40; *n* = 35 terminals; Fig. 4*B*). Overall, the results are similar to those of Orr et al. (2014), who used slightly different methods and reported that 10.93% of the control terminal volume was occupied by gap junctions and 3.00% of the terminal in *frazzled* LOF specimens.

Since latency and gap junction antibody volume have been linked directly in the GFS (Augustin et al., 2017), we compared the latency averages and the proportion of volume taken up by gap junction antibody in GF terminals for each sample. To compare the various genotypes, we ran a KS2D2S test comparing the latency (and, separately, the response frequency) averages to the average proportion of gap junction antibody volume in unit terminals. By comparing each experimental group to a control group using the KS2D2S test, we perform separate, independent tests and avoid raising the familywise error rate. In Table 1, we show the results of the KS2D2S test done comparing the *frazzled* LOF mutant (Sample 1 column, *fra*^3^/*fra*^4^) and all our tested UAS constructs (Sample 2 column). We listed the number of terminals compared for each UAS construct in a separate column. For example, in the first row of Table 1, we compared *frazzled* LOF mutants and their control siblings (*fra*^3^/+). We found significant differences in the distribution of latency averages to the proportion of volume taken up by gap junction antibody in GF terminals for our samples (*p* = 0.0001; Table 1). Similarly, response frequency and gap junction volume were significantly different (*p* = 0.0001; Table 1). These results strongly suggest that the *frazzled* LOF is associated with gap junction loss at the GF terminals.

### Bilateral terminals are an indirect result of *frazzled* LOF

Bilateral terminals appeared in all mutant genotypes examined and were not observed in wild-type specimens (Fig. 1*B*,*H*). Visual inspection and summary statistics of the three physiological measures (latency average, gap junction antibody, 100 Hz average) did not reveal noticeable functional differences between *frazzled* LOF terminals that were wild-type-appearing versus those that were bilateral. We applied multivariate and dimension-reduction methods to formally test whether these two categories could be treated as a single functional group. Because the three measures are correlated, we performed PCA on the standardized measures to capture shared variance. We also extracted individual PC scores for each terminal (see Materials and Methods). For the w; *fra^3^*/*fra^4^*; R91H05::GFP/+ genotype, where both wild-type-appearing and bilateral subgroups were sufficiently represented, a MANOVA on PC1–PC3 showed no multivariate difference between the groups (*F* = 0.261; *p* = 0.853), and univariate comparisons of PC1–PC3 were also nonsignificant (PC1, *t* = −0.784; *p* = 0.439; PC2, *t* = −0.041; *p* = 0.968; PC3: *t* = −0.438; *p* = 0.664); after correction for the three within-genotype comparisons (Bonferroni, Holm, and Benjamini–Hochberg), none of the PCs were significant (adjusted *p* ≥ 0.968). Because these multivariate and corrected univariate analyses indicate no detectable functional separation between wild-type-appearing and bilateral terminals in this genotype, we treated each terminal as a single analysis unit. We combined wild-type-appearing and bilateral terminals for the analyses reported in the results.

Unit terminals from wild-type-appearing GFs exhibit a latency of 1.14 ms (SD, 0.32; *n* = 20 terminals; Fig. 4*A*) and response frequency of 79.60% (SD, 25.73; *n* = 20 terminals; Fig. 4*D*), while the unit terminals from bilateral GFs exhibit a latency of 1.23 ms (SD, 0.25; *n* = 13 terminals; Fig. 4*A*) and a response frequency of 51.54% (SD, 29.50; *n* = 13 terminals; Fig. 4*D*). In a *frazzled* LOF wild-type-appearing unit terminal and bilateral unit terminal, gap junction antibody occupied 5.54% of wild-type-appearing GF terminals (SD, 2.29; *n* = 20 terminals) and 4.61% of the bilateral unit terminals (SD, 2.51; *n* = 16 terminals).

To better understand the bilateral terminals in the context of *frazzled*, we created bilateral terminals in wild-type animals by laser ablation of one GF and analyzed the remaining GF, which nearly always (13/15) produced a bilateral terminal (Boerner et al., 2024). We compared these ablation-induced bilateral terminals (latency, response frequency, and proportion of the synaptic terminal occupied by the antibody to gap junctions) with bilateral terminals in *frazzled* LOF mutants (Table 1). Bilateral terminals created in a wild-type background exhibit wild-type proportions of gap junction antibody volume in the unit terminals (9.65%; SD, 5.76; *n* = 22 terminals; Fig. 4*B*). This suggests that the single GF is producing enough synaptic material to supply both branches of the bilateral terminal and therefore producing twice the amount of gap junction as a normal GF.

The comparison of bilateral terminals created by ablation to those caused by the *frazzled* LOF mutant highlights the role of Frazzled in gap junction assembly. The ablation in a wild-type background produced bilateral terminals with wild-type amounts of gap junction, while the bilateral terminals in a *frazzled* LOF mutant background have less gap junction in the terminal. This makes two points: (1) the *frazzled* gene regulates gap junction levels, but (2) it does not regulate the terminal growth when the contralateral neighbor is missing. The lack of competition seems to have the same effect in mutant and control backgrounds; an extra branch grows to form a bilateral terminal completely independent of the Frazzled receptor.

### Full-length UAS-Frazzled partially rescues the GF electrical synapse

To refine our idea that the ICD of Frazzled controls the assembly of the electrical synapse, we dissected the Frazzled receptor using the UAS-GAL4 system. We drove several different UAS-Frazzled constructs (Fig. 1*I*; Neuhaus-Follini and Bashaw, 2015) in a *frazzled* LOF background and assayed the ability of a given construct to rescue the LOF phenotypes.

First, we drove full-length UAS-Frazzled in the GFs of *frazzled* LOF mutants using the GF driver R91H05-GAL4 recombined with GFP to label the GF axons (w; *fra*^3^/*fra*^4^; R91H05::GFP-GAL4/UAS-Frazzled; Fig. 1*I*; Borgen et al., 2017; Jenett et al., 2012; Orr et al., 2014). We analyzed the physiology of 20 *frazzled* LOF flies where UAS-Frazzled was driven in the GFs and found they had response latencies averaging 1.07 ms (SD, 0.20; *n* = 38 terminals; Fig. 4*A*) and response frequencies averaging 87.17% (SD, 22.90; *n* = 38 terminals; Fig. 4*D*). We generated 3D volume renderings to assess the volume of anti-shaking-B gap junction antibody present within each unit terminal of LOF flies driving (Fig. 3*D*,*E*). We compared response latency and the proportion of volume taken up by gap junction antibody in GF terminals of UAS-Frazzled and *frazzled* LOF mutants. We found a significant difference between the distributions for our samples using a KS2D2S test (*p* = 0.0373; Table 1). A similar difference was found when comparing response frequency and proportion of volume taken up by gap junction antibody in GF terminals (*p* = 0.0076; Table 1). This rescue of synaptic structure and function agrees with prior findings (Orr et al., 2014) and strongly supports our hypothesis that Frazzled regulates synaptogenesis in the GFs.

### The ICD of Frazzled rescues GF synaptic structure and function

Frazzled’s ICD is known to be involved in several intracellular signaling pathways (Boyer and Gupton, 2018), so we next attempted to rescue the LOF phenotypes with the Frazzled ICD. We analyzed the GF anatomy of 23 flies and found a partial rescue of the axon guidance phenotypes. Seventeen of the flies had wild-type-appearing GFs, six had one GF stuck in the brain, and none had both GFs stuck in the brain (Fig. 1*H*). We analyzed the latency, response frequency, and proportion of volume taken up by gap junction antibody in GF unit terminals of 12 flies and found they exhibited wild-type responses and had an average response latency of 0.97 ms (SD, 0.06; *n* = 23 terminals; Fig. 4*A*) and a response frequency average of 98.40% (SD, 3.16; *n* = 23 terminals; Fig. 4*D*). A KS2D2S test between the *frazzled* LOF mutants and mutants driving Frazzled ICD found a significant difference between the distributions of response latency and response frequency averages to the proportion of volume taken up by gap junction antibody in GF terminals (*p* = 0.0007; *p* = 0.0004; Table 1). These findings demonstrate that the Frazzled ICD nearly completely rescues all the LOF mutant phenotypes independent of Frazzled’s extracellular domain.

### Deleting the conserved ICDs (P1, P2, or P3) alters rescue outcomes

To further investigate what part of the ICD is essential for GF structure and function, we tested three constructs that express full-length Frazzled, each missing one of its highly conserved ICDs: UAS-FraΔP1, UAS-FraΔP2, and UAS-FraΔP3 (Fig. 1*I*; Neuhaus-Follini and Bashaw, 2015). Driving these constructs individually in *frazzled* LOF backgrounds revealed the rescue of distinct GF terminal phenotypes for the different domains. For UAS-FraΔP1, we analyzed 13 flies and found none had wild-type-appearing terminals, six had bilateral terminals, and seven had both GFs stuck in the brain (Fig. 1*H*). Latencies averaged 1.80 ms (SD, 0.61; *n* = 7 terminals; Fig. 4*A*), response frequencies averaged 54.05% (SD, 29.14; *n* = 7 terminals; Fig. 4*D*), and gap junction antibody made up 7.65% of the unit terminal volume on average (SD, 2.53; *n* = 7 terminals; Fig. 4*B*). Here, the KS2D2S showed that the results for latency, response frequency, and gap junction volume were different from LOF, implying a partial rescue of the gap junctions (*p* = 0.0013; *p* = 0.0153; Table 1). Similarly, for UAS-FraΔP2, we analyzed 10 flies and found two had wild-type-appearing terminals, eight had bilateral terminals, and none had both GFs stuck in the brain (Fig. 1*H*). Latencies averaged 1.40 ms (SD, 0.25; *n* = 20 terminals; Fig. 4*A*), response frequencies averaged 54.05% (SD, 29.14; *n* = 20 terminals; Fig. 4*D*), and gap junction antibody in the unit terminals occupied 8.26% of the unit terminal volume on average (SD, 3.85; *n* = 20 terminals; Fig. 4*B*). Similar to ΔP1’s KS2D2S result, flies driving UAS-FraΔP2 also showed significant differences compared with LOF flies (*p* = 0.0002; *p* = 0.0054; Table 1). These results suggest a partial rescue of the gap junctions using these constructs (UAS-FraΔP1 and UAS-FraΔP2).

In contrast to UAS-FraΔP1 and UAS-FraΔP2, we analyzed 20 UAS-FraΔP3 flies and found seven had wild-type-appearing terminals, eight had bilateral terminals, and five had both GFs stuck in the brain (Fig. 1*H*). Here, response latencies averaged 1.46 ms (SD, 0.69; *n* = 11 terminals; Fig. 4*A*), response frequencies averaged 60.00% (SD, 31.67; *n* = 11 terminals; Fig. 4*D*), and gap junction antibody occupied 5.37% of the unit terminal volume on average (SD, 1.41; *n* = 11 terminals; Fig. 4*B*). The KS2D2S test showed UAS-FraΔP3 is not different from LOF, demonstrating the absence of any rescue effect on the synapse (*p* = 0.3085; *p* = 0.5607; Table 1). Taken together, the data suggest that the P3 domain of Frazzled is required for regulating gap junctions in the GF, while P1 and P2 are not necessary.

### The Frazzled transcriptional activation domain is required to rescue synaptic function

To further dissect Frazzled’s ICD, we drove a full-length Frazzled construct containing a point mutation in the P3 domain in a LOF background (Fig. 1*I*; Fig. S1). This ICD mutation (FraE1354A) is known to prevent transcription of commissureless, a midline guidance protein expressed during larval development (Neuhaus-Follini and Bashaw, 2015). When we analyzed the GF terminals of LOF flies driving UAS-HA-FraE1354A, we found that this construct rescued neither axon guidance nor synaptogenesis. Three flies exhibit wild-type-appearing terminals, six flies with bilateral terminals, and five with both GFs stuck in the brain (Fig. 1*H*). When we analyzed the physiology of UAS-HA-FraE1354A flies, we found they had an average latency of 1.33 ms (SD 0.60; *n* = 13 terminals; Fig. 4*A*) and a response frequency average of 52.69% (SD 38.72; *n* =13 terminals; Fig. 4*D*), and gap junction antibody occupied 4.18% of the unit terminal volume on average (SD, 2.75; *n* = 13 terminals; Fig. 4*B*). When we compared the distributions of response latency and response frequency averages each to the proportion of volume taken up by gap junction antibody in unit terminals of UAS-HA-FraE1354A flies and *frazzled* LOF mutants with a KS2D2S test, we found no significant difference between the response latency and average proportion of gap junction antibody volume but did find a difference between response frequency and proportional gap junction antibody (*p* = 0.0628; *p* = 0.0404; Table 1). The average latency, response frequency, and proportion of gap junction antibody in unit terminals are significantly worse when we drive UAS-HA-FraE1354A flies in a *frazzled* LOF background compared with *frazzled* LOF alone. We also tested flies with one copy of the construct driven in a wild-type background and found no significant difference between them and wild-type flies with no construct driven, confirming that the construct itself does not cause these phenotypes (*n* = 12; average latency = 0.86 ms; SD, 0.085; *p* = 0.4714; average response frequency = 99.81%; SD, 0.46; *p* = 0.6784). Taken together, this finding suggests that UAS-HA-FraE1354A produces a dominant negative effect on the *frazzled* LOF mutant, strongly supporting our hypothesis that Frazzled's transcriptional activation domain is required to rescue gap junctions in a *frazzled* LOF mutant background.

### Modeling the role of gap junctions in synaptic function

As the amount of gap junction was reduced in the experimental animals, stimulus response failures and successful response latency both increased (Fig. 4*A*,*C*,*D*). To explore the role of gap junctions in response decrement, we created a model of the GF–TTMn circuit and simulated the amount of gap junctions available at the virtual synapse in a manner that parallels the experimental data with *frazzled* mutants. Our primary goal was to determine how gap junction-controlled circuit function, namely, the latency and response decrement of the GF→TTMn synapse in our various genotypes.

**Figure 5.**
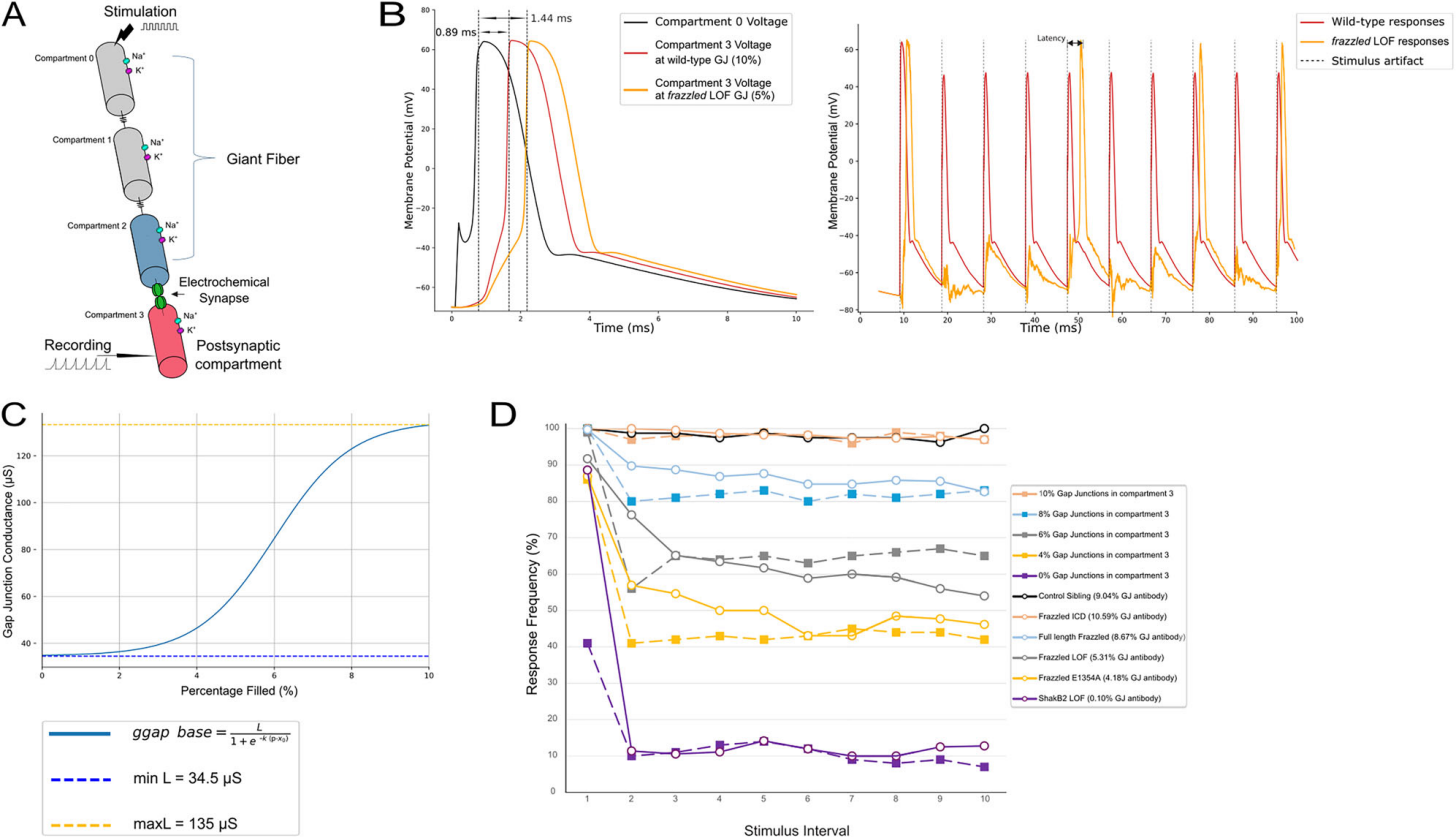
Modeling the GF→TTMn synapse effectively simulates responses to stimulation when altering gap junction proportions in terminals. ***A***, Our model uses compartments to simulate the GF System. A stimulus is introduced in Compartment 0 and is propagated through subsequent compartments that make up the GF model. Between Compartments 2 and 3, we placed a simulated electrochemical synapse and varied the amount of gap junction present at the synapse to alter responses to stimulation. An action potential is then produced in Compartment 3, where response latency and frequency are recorded. ***B***, Top panel, The recordings from Compartment 3 can be seen for wild type in red and *frazzled* LOF in orange. Latency is calculated as the difference in time between the peaks of Compartment 0, shown in black, and Compartment 3. ***B***, Bottom panel, Response frequency is measured by the number of action potentials recorded for 100 Hz stimulation wild-type (red) and *frazzled* LOF (orange). ***C***, Gap junction conductance in the model is modulated by a sigmoid transformation. In wild-type flies, the amount of gap junction protein present in the GF terminals is, on average, 10% of the total volume of the terminal. In a *shak-B*^2^ mutant, gap junctions are absent from the GF→TTMn synapse, and the GF relies solely on the chemical synapse for signaling (Boerner et al., 2024). Gap junctions are naturally lost as the fly ages, so we set our limits of gap junction conductance following parameters found in Augustin et al. (2019) that follow age-related decreases in conductance. We added an exception for zero gap junctions, which led to zero conductance. We used a sigmoid equation to simplify the Boltzmann equation since we want to look at the effects of varying gap junction proportions on responses and not channel dynamics. ***D***, We find that our model (dashed lines) can closely mimic the response decrement profile of our *frazzled* mutant flies (solid lines). Colors as in Figure 4.

We first developed a compartment model of the GFs that mimicked the signaling properties of the GF and then modeled the electrochemical synapse to connect the model GF to a postsynaptic compartment. We adapted a model previously used to examine gap junctions during aging (Augustin et al., 2019) and used parameters from the Augustin model to represent the features of our compartment model GF. We then reduced the amount of gap junction in the model in a manner that paralleled the results from our various *frazzled* genotypes. The basic properties of the model are shown in Figure 5. A signal is introduced in the furthest compartment from the synapse (gray) and propagates into connected compartments until it reaches the synapse (blue), where the electrochemical dynamics determine if an action potential is produced.

The amount of gap junction observed in our experimental data was used to set the range of gap junction function in the model. The volume of anti-gap junction antibody in a control terminal is ∼10% of the terminal volume and 5.31% in the mutant terminal (Fig. 4*B*). We used *shak-B^2^* flies as the lower boundary of gap junction antibody, which was shown to be ∼1% of the terminal (Boerner et al., 2024). We applied a sigmoid transformation to simplify the conductance-per-gap junction amount calculation and set the upper and lower limits of conductivity based on parameters found in the Augustin et al. (2019) model (Fig. 5*C*).

The model exhibited latency increases as the amount of gap junction was reduced (Fig. 5*B*). This was expected and consistent with the Augustin et al. (2019) data. What was somewhat surprising was that the rate of response decrement increased as the proportion of gap junction was reduced (Fig. 5*D*, dashed lines). Since gap junctions are not typically thought to show activity dependence, the source of this response decrement was unclear and not explored by Augustin et al. (2019). Using the volume of gap junction antibody as a proxy for gap junction function, we simulated the model's response at different proportions of gap junction seen in various genotypes. The model accurately simulates the response decrement as shown by the data from multiple genotypes [Fig. 5*D*, dashed lines (simulations) and solid lines (experimental data)]. Reducing the gap junction proportion to zero dramatically reduced the response, leaving a very weak response, as seen in *shak-B^2^* mutants with no functional gap junctions (Fig. 5*D*). Removal of the chemical synapse in the model did not significantly alter synaptic function, as in the wild-type animal.

## Discussion

The results reinforce prior findings and provide novel mechanistic insight into how Frazzled regulates synaptogenesis in the GFs. Specifically, we show that intracellular regions of Frazzled regulate the presence of presynaptic gap junctions shaking-B(neural+16) that make up the electrical synapse of the GF. We quantified synaptogenesis by using response latency, response decrement, and the amount of gap junction antibody in the GF terminals. By combining electrophysiological data and confocal imaging, we show strong evidence of a role for Frazzled in regulating electrical synapses in the GFs. Simulation studies demonstrated how this control of gap junctions regulates function at the synapse.

### Frazzled regulates synaptic structure and function in GFs

In *frazzled* LOF mutant flies, the GFs exhibit long response latencies and rapid response decrement due to the reduced density of gap junctions (Fig. 4*A*,*C*; Table 1; Orr et al., 2014). We focused on the presynaptic side of the GF→TTMn electrical synapse, which is assembled from the shaking-B(neural+16) isoform (Phelan et al., 2008). Our shaking-B antibody results show that shaking-B gap junctions make up 9.04% of GF terminal volume in control siblings and 5.14% of GF terminal volume in LOF mutants (Fig. 4*B*).

### The Frazzled ICD rescues synaptogenesis

To analyze Frazzled’s synaptogenic role in more detail, we attempted to rescue synaptic function in LOF mutants by driving various fragments of the Frazzled receptor in the LOF background (Fig. 1*I*; Neuhaus-Follini and Bashaw, 2015). The most dramatic result occurred when we drove a UAS construct of the highly conserved Frazzled ICD (UAS-FraICD; Fig. 1*I*). This construct rescued most of the LOF phenotypes, including all the synaptogenic phenotypes (Fig. 1*H*, 4; Table 1). The results show response latency averages, response decrement, and the proportion of volume occupied by gap junction antibody in GF terminals flies carrying the ICD rescue construct are restored to wild-type levels (Fig. 4).

The dramatic rescue effects of the ICD led us to examine other variations of the Frazzled receptor focused on the ICD to better understand its function. The ICD contains a transcriptional activation domain overlapping the Frazzled P3 domain (Neuhaus-Follini and Bashaw, 2015). To examine the role of the P3 domain in a synaptic context, we drove a full-length Frazzled construct, lacking the P3 domain (UAS-FraΔP3; Fig. 1*I*) in the *frazzled* LOF background and assessed synaptic structure and function. This construct did not rescue the synaptic defects in the LOF mutants (Table 1).

To extend the analysis of the P3 domain, we drove a full-length Frazzled construct with a point mutation in the highly conserved P3 domain that is known to silence the transcriptional activation domain in the context of axon pathfinding in embryos (UAS-FraE1354A; Fig. 1*I*; Neuhaus-Follini and Bashaw, 2015). In this rescue experiment, the physiology and proportion of gap junction antibody in terminals were not different from those seen in *frazzled* LOF mutant flies (Table 1). These two constructs are known to affect transcription of *commissureless* in embryos (UAS-FraΔP3 and UAS-FraE1354A; Neuhaus-Follini and Bashaw, 2015), and we suggest that Frazzled’s transcriptional activation domain is similarly required for gap junction synthesis in the GFs. We hypothesize that Frazzled’s transcriptional activation domain regulates transcription of the innexin shaking-B(neural+16).

The role of Frazzled/DCC homologs in chemical synapse formation and function has been described in *C. elegans* with interesting parallels and contrasts to *Drosophila*. In an early study of the role of glia in synapse formation secretion of Netrin by glia was shown to regulate chemical synaptogenesis via the UNC-40/DCC pathway (Colon-Ramos et al., 2007). More recent efforts have demonstrated that both GABAergic and cholinergic chemical synapse are regulated by UNC-40/DCC (Zhou and Bessereau, 2019; Zhou et al., 2020). We draw a parallel to our work in *Drosophila* through the critical role the P3 domain plays in the assembly of GABAergic synapses. In *C. elegans*, deletion of the P3 domain was critical to clustering of GABA_A_ receptors and less critical to the guidance phenotypes. In *Drosophila*, the P3 domain was critical to gap junction formation but less critical to axon guidance. We interpreted this as a specific, transcriptional effect of the Frazzled ICD in flies as the critical transcription sequence found in Frazzled’s P3 domain (E in E1354A) is not present in homologous UNC-40's P3 domain (Altschul et al., 1997).

### The role of Frazzled in GF axon guidance

Our primary interest in this work was the control of synapse formation and function. However, Frazzled is a well-known axon guidance molecule, and we observed several disruptions of axon guidance linked to Frazzled. In the *frazzled* LOF animals, nearly half of the GFs exhibit severe guidance defects, grow randomly in the brain, never reach the thorax, and do not make synaptic connections with their normal targets. When full-length Frazzled was driven in the LOF mutants, the trends in the rate of GF axon pathfinding errors decreased overall but were not significantly different from *frazzled* LOF mutants. We especially noted no change in the frequency of stuck-in-the-brain pathfinding errors. In contrast to the full-length construct, driving the ICD in the LOF mutants nearly completely rescued these stuck-in-the-brain defects. The strong effects of the ICD led us to examine the role of the ICD in axon guidance in more detail. We drove full-length constructs lacking one of the three domains (ΔP1, ΔP2, or ΔP3) in *frazzled* LOF mutants (Fig. 1*I*). The constructs lacking the ΔP1 and ΔP2 domains did not rescue axon guidance defects but did rescue the physiology and the gap junction antibody volume. In contrast, the ΔP3 construct and the point mutation in the presumed transcriptional activation domain did not rescue the axon guidance phenotypes nor the synaptic defects (Fig. 1*H*; Table 1). This last result supports our suggestion that Frazzled regulates gap junction through its transcriptional activation domain.

### A computational model of the GF→TTMn synapse

We developed a computational model of the GFS in *Drosophila* as a tool to assess the function of the GF System in the various mutant phenotypes (Fig. 5*A*,*B*). We used a conductance-based, multicompartmental model with ionic currents derived from the GF (Augustin et al., 2019). A novel aspect of our model is the sigmoidal function that dynamically adjusts gap junction conductance based on the volume of the terminal occupied by gap junctions (Fig. 5*C*). The rationale behind sigmoid functions comes from the previous use of Boltzmann functions to model the typical nonlinear conductance change between two values (Moreno et al., 1995; Valiunas et al., 1999). This approach allows for precise control of synaptic strength, making the model particularly suited for studying the effects of reduced gap junctions, as observed in *frazzled* LOF mutants.

The model highlights the strong relationship between following frequency and gap junction volume in the GF terminal. We ran the model using different volumes of gap junction in the GF terminal corresponding to various genotypes and generated a range of response frequency curves (Fig. 5*D*). We find that the gap junction antibody proportion of flies nicely predicts their response frequency data. We compared our results to data for a mutation (*shak-B^2^*) that silences gap junctions in the GFS (Phelan et al., 2008). In this experiment, mutant flies lacking functional gap junctions were partially rescued by driving the gap junction gene shaking-B(neural+16) in the *shak-B^2^* LOF background. The results of these rescue experiments with *shak-B^2^* were very similar to our results with Fra/DCC. The range of response frequency data from these *shak-B^2^* experiments fit nicely into our model’s range of response frequency curves. Using a relatively strong GF driver (A307) or a weaker driver (C17) to express shaking-B(neural+16) the response frequencies place the likely percent of their terminal occupied by gap junctions between 4 and 7% (Phelan et al., 2008). This is parallel to our experiment where Fra/DCC regulates the gap junction proportions over a similar range, and rescue constructs result in GF responses very similar to the model and to the Phelan et al. (2008) results.

The model incorporates periodic inputs with Gaussian noise to mimic biological variability, providing a realistic simulation of external stimuli affecting the GFs and synaptic failure in its transmission. However, the model also has limitations that warrant consideration. One such limitation is the simplification of certain aspects of synaptic dynamics, such as terminal morphology. The model is designed under the implication that a wild-type GF terminal connection to the TTMn is made. When terminals are bilateral or otherwise deformed, the distribution of gap junctions and other synaptic proteins changes. We can model such changes indirectly changing signaling dynamics in the model, but the exact changes happening in vivo are not fully understood. However, the flexibility of the model allows for subcompartments to be created, mimicking bilateral morphology. Each subcompartment can then be modified with their own gap junction proportions and chemical signaling changes to reflect the bilateral nature of the GF terminal. We may still learn something from these future expansions of the model to include bilateral terminals. Future refinements to the model will allow us to simulate synaptic connections beyond the traditional GFS and will let us build a model of the escape reflex from sensory neuron to motor neuron and every interneuron in between.

## Synthesis

Reviewing Editor: Mei Zhen, University of Toronto Decisions are customarily a result of the Reviewing Editor and the peer reviewers coming together and discussing their recommendations until a consensus is reached. When revisions are invited, a fact-based synthesis statement explaining their decision and outlining what is needed to prepare a revision will be listed below. The following reviewer(s) agreed to reveal their identity: Ben Mulcahy. Note: If this manuscript was transferred from JNeurosci and a decision was made to accept the manuscript without peer review, a brief statement to this effect will instead be what is listed below.

Synthesis: This manuscript advances our understanding of how the guidance molecule Frazzled/DCC influences gap junction formation, potentially through mechanisms independent of its canonical role in axon guidance. The use of domain-specific rescue constructs adds mechanistic insight into how specific intracellular regions of Frazzled/DCC contribute to synapse assembly. In particular, the intracellular domain appears to play a key role. The authors provide suggestive evidence - based on a point mutation in the transcriptional activation domain - that this effect may be mediated at the transcriptional level, although no direct transcriptional analyses are performed. Overall, the study contributes to a growing body of evidence that axon guidance receptors have broader functions in synapse development, including the formation of electrical synapses.

Reviewer's comments: Experimentally, there is convincing evidence that the Frazzled intracellular domain is important for GJ formation. It is intriguing that FraE1354A fails to rescue in a Frazzled LOF genotype, suggesting a role for Frazzled-dependent transcriptional regulation of GJ formation. However, anti-HA staining is necessary to validate that FraE1354A is properly expressed.

I found this manuscript difficult to read for the following reasons:

1) The writing is unclear in places - it could do with another round of edits to improve clarity and conciseness

2) The manuscript appears to be written for a specialist audience and may not be accessible for the broader neuroscience readership of eNeuro

3) There are inconsistencies distributed across the writing. For example, Flamingo is randomly referred to as 'Flamingo', 'flamingo', 'Flamingo/DCC', there is a mixture of 'wildtype' and 'wild-type', Drosophila is not always italicized

I also found that the discussion section largely reiterates the results without deeper interpretation or contextualization to the broader literature.

Discussion of the role of Frazzled in chemical synaptogenesis is missing, along with relevant references including:

Horn et al. (2013) DCC Expression by Neurons Regulates Synaptic Plasticity in the Adult Brain, Cell Reports

Tu et al. (2015) C. elegans Punctin Clusters GABAA Receptors via Neuroligin Binding and UNC-40/DCC Recruitment, Neuron

Zhou et al. (2020) The netrin receptor UNC-40/DCC assembles a postsynaptic scaffold and sets the synaptic content of GABAA receptors, Nature comm.

Specific points below:

Line 13: 'Frazzled fails to rescue the synaptic function' - the 'the' should not be there

Line 29-30: 'The present work is the first to show how a guidance molecule regulates synaptogenesis through transcriptional regulation of synaptic components' - no it is not. A reviewer brought this up in the last round of review - this needs to be corrected

Line 39: A past reviewer made the point that you should credit Hedgecock and colleagues for discovering netrin, but you have the wrong reference. Should be Hedgecock, Culotti and Hall (1990)

Lines 53-54: 'This synapse has been studied since the early 1980s, and the invertebrate gap

junctions (GJs) were discovered and characterized there (Blagburn et al., 1999; Phelan et

55 al., 2008; Thomas &Wyman, 1983).' - this writing is unclear - it may be read as if you are suggesting invertebrate GJs were originally discovered at the GF-TTMn synapse - which of course is not the case.

Line 57: 'mediated by the innexin gene for gap junctions' - awkward phrasing that is vague and imprecise

Lines 59-60: 'Shaking-B gap junctions are composed of four protein subunits in the presynaptic and four in the postsynaptic membranes (Oshima, 2020; Phelan et al., 2008)' - this is incorrect. Each innexin has four transmembrane helices, but each hemichannel is composed of six subunits. Also the Oshima opinion paper is out of place - it makes no mention of shaking-B

Line 79-80: 'Frazzled may be regulating innexin GJs in the GFs at the transcriptional level and we dissected the Frazzled/DCC receptor' - awkward and redundant use of 'innexin GJs', and the ending does not make sense

Line 84: 'We demonstrate that Frazzled's ICD is necessary and sufficient to produce the GF-specific gap junctions shaking-B(neural+16) by quantifying...' - it doesn't quite make sense. Are you saying shaking-B(neural+16) GJs are specific to GFs? Did you mean to write 'gap junctions formed with shaking-B'? Even GF-specific gap junctions doesn't make sense, because the GJs are formed between GFs and TTMns.

Lines 129-131: 'Approximately half of the GF axons grow aberrantly in the brain and never reach the ventral nerve cord. The remaining...' - in the mutants or in wild-type?

Line 154-155: 'Consequently, each principal component was then compared between WT and Bilateral individuals using t-tests.' - were there corrections for multiple comparisons?

Line 162: 'the head case was also opened to reveal the brain' - expose the brain?

Lines 166-167: Could the authors state in the text where the anti-shaking-B antibody binds and how it relates to the different isoforms

Lines 178-179: 'Samples were magnified using a 60x oil immersion objective scanned at a pixel resolution of 1024 x 1024, and z-step sizes were 0.125μm.' - it is odd (and not useful to readers) to report the pixel dimensions, especially when the z-step sizes are in µm.

Lines 180-181: 'contrast and brightness were adjusted to increase image quality' - the image quality remains the same - you are just stretching the intensities to change how it is displayed

Lines 187-189: 'We applied a gap junction antibody mask to assess the volume in the GF terminals taken up by the gap junction antibody channel mask to show only the GF terminal content, excluding the antibody label outside the terminal.' - the wording is unclear. Also please describe how the mask was generated

Lines 205-206: please give a short description contained within the methods section of how you ablated

Lines 211-214: describe how you detected neurobiotin. Also where you purchased it

Line 286: 'using various UAS-Frazzled constructs' - another example of vagueness

Line 351: 'Bilateral terminals appeared in most genotypes in these experiments' - excluding wild-type?

Lines 352-353: 'When we separate the terminals found in Frazzled LOF specimens into two groups (wild-type-appearing and bilateral), they are functionally indistinguishable' - the description of the PCA comes later in this section, leaving the reader confused at this point because this statement appears unjustified.

Line 395: 'Full-length UAS-Frazzled rescues the Giant Fiber electrical synapse' - it partially rescues

Lines 415-416: 'This rescue of synaptic structure and function strongly supports our hypothesis

that UAS-Frazzled regulates synaptogenesis in the GFs.' - this is presented as a novel hypothesis, but is already well established by the work of the authors and others. Also, why UAS-Frazzled?

Lines 474-475: 'When we analyzed the GF terminals of LOF flies driving UAS-HA-FraE1354A,

we found that this construct rescued neither axon guidance nor synaptogenesis' - did the authors stain with anti-HA to verify the protein is expressed?

Lines 490-491: 'We also tested flies with one copy of the construct in a wild-type background

and found no significant difference between them' - where is the data?

Lines 497-499: 'As the amount of gap junction was reduced in the experimental animals, the latency and rate of response decrement both increased (Figure 4A, 4C and 4D). Since gap junctions are not typically use-dependent, the increased rate of response decrement (Figure 4C) was an enigma.' - the authors use the phrase 'response decrement' which I think refers to less reliability/greater latency. I find the phrasing imprecise. Agree GJs are not typically use-dependent - but include citation. Calling it an 'enigma' is unusual for the results section of a scientific paper, but if the authors want that then fine.

Line s 504-505: 'One prominent feature of function is the dramatic response decrement induced in multiple genotypes exhibiting different levels of gap junction in the terminals' - I'm not sure what you mean by 'one prominent feature of function' - it comes across as nonsensical

Lines 542-543: 'The results confirm our hypothesis that Frazzled/DCC regulates synaptogenesis in the Giant Fibers by regulating gap junctions' - this is presented as a novel hypothesis, but is already well established by the work of the authors and others

Lines 546-548: 'By combining electrophysiological data and confocal imaging, we show how Frazzled regulates the assembly of electrical synapses.' - this is a misleading overstatement. The authors show that the frazzled intracellular domain is able to rescue, to some degree, the loss-of-function phenotypes, and a construct with a point mutation in the transcriptional activation domain is unable to rescue, suggesting something to do with transcription.

Line 555: 'antibodies to gap junctions' - be specific

Lines 730-731: 'Model Author. (2025). XXXXXXXXXXXXXXXXXXXXXXXXXXXXXXXX

[Computer software]. https://doi.org/00.0000/0000.00.00.647628'

Line 812: 'Figure 1. Axon guidance phenotypes and resulting physiology' - 'physiology' is used throughout the paper and is vague

Lines 826-827: 'UAS-FraE1354A contains an HA tag that does not influence synaptic function (not shown).' - show it

Line 841: 'Compressed z-stack projection image' - what kind of z-stack? Maximum intensity? Average intensity? Other?

Lines 842-843: 'shaking-B(neural+16) gap junctions labeled with shaking-B antibody' - does the antibody bind specifically to neural+16 or to all shaking-B innexins?

Figure 1 A-G: it would be much easier for readers if the genotype was labelled on the panels

Figure 1H-I: The orders of the genotypes are mixed up between H and I, making it harder to read. Also in panel I showing the different proteins expressed by the different constructs, the UAS is not particularly relevant

Figure 2C and Figure 3 right panels: scale for recordings

Figure 3: a cartoon of the Frazzled protein variant would be a useful addition

Figure 4C - axis labels?

## Author Response

Dear Dr Zhen, We appreciate the excellent review of our manuscript. We found it to be thorough, detailed, and very useful. We have addressed each of the comments in bullet points throughout the review below. The line numbers correspond with the line numbers found in the Article File.

-------------------------------------------- Manuscript Instructions The claim of 'transcription' was not fully removed in the revised manuscript (e.g. the running title; the text - see Specific comment below). • This is now corrected throughout the submission.

--------------------------------------------- Synthesis of Reviews:

Computational Neuroscience Model Code Accessibility Comments for Author (Required):

The authors provide a Jupyter Notebook with their computational model of the GF-TTMn synapse. The notebook has several significant issues:

1) The notebook does not reproduce the results shown in Figure 5 of the manuscript. The output plots do not clearly correspond to the published data. • The notebook now produces plots found in Figure 5. Some minor editing was performed for visual clarity outside of the code (color changes, spacing).

2) Upon re-running the notebook, the plots generated differ from those originally saved in the notebook. This suggests that the code has changed since it was last executed, raising concerns about reproducibility. • This is now corrected, and the code reproduces the figures on every run.

3) Multiple plotting cells appear to generate similar plots with differing titles, and it is unclear how they relate to the final figures in the manuscript. • These have been removed entirely.

4) The notebook includes empty code cells at the end, yet saved plots still appear, indicating that some code has been removed. There appears to be a reference to channelrhodopsin despite no related simulation code being present. • This is also entirely removed from the code.

5) The code is poorly documented • The code now contains significantly more documentation of each function.

The notebook needs substantial cleanup and annotation. At a minimum, it should (a) reproduce the figures in the manuscript, (b) match saved plots to live code, (c) remove orphaned output, and (d) include documentation sufficient for an external user to follow and test the model.

Synthesis Statement for Author (Required):

Synthesis: This manuscript advances our understanding of how the guidance molecule Frazzled/DCC influences gap junction formation, potentially through mechanisms independent of its canonical role in axon guidance. The use of domain-specific rescue constructs adds mechanistic insight into how specific intracellular regions of Frazzled/DCC contribute to synapse assembly. In particular, the intracellular domain appears to play a key role. The authors provide suggestive evidence - based on a point mutation in the transcriptional activation domain - that this effect may be mediated at the transcriptional level, although no direct transcriptional analyses are performed. Overall, the study contributes to a growing body of evidence that axon guidance receptors have broader functions in synapse development, including the formation of electrical synapses.

Reviewer's comments: Experimentally, there is convincing evidence that the Frazzled intracellular domain is important for GJ formation. It is intriguing that FraE1354A fails to rescue in a Frazzled LOF genotype, suggesting a role for Frazzled-dependent transcriptional regulation of GJ formation. However, anti-HA staining is necessary to validate that FraE1354A is properly expressed. • We have included anti-HA staining as Supplemental Figure 1.

I found this manuscript difficult to read for the following reasons:

1) The writing is unclear in places - it could do with another round of edits to improve clarity and conciseness • We have revised portions of the text that were identified in the line-by-line review for clarity.

2) The manuscript appears to be written for a specialist audience and may not be accessible for the broader neuroscience readership of eNeuro • We introduced and modified existing language in key parts of the text to make the findings and discussion more accessible.

3) There are inconsistencies distributed across the writing. For example, Flamingo is randomly referred to as 'Flamingo', 'flamingo', 'Flamingo/DCC', there is a mixture of 'wildtype' and 'wild-type', Drosophila is not always italicized • Although 'flamingo' is never referred to in this manuscript, we have nevertheless standardized the usage of Frazzled following conventional wisdom. Frazzled for proteins, frazzled for genes and gene mutations, and Frazzled/DCC is limited to introducing the protein family, as it is commonly addressed as such. We also stick to 'wild-type; over wildtype, and made sure Drosophila remains italicized throughout the text.

I also found that the discussion section largely reiterates the results without deeper interpretation or contextualization to the broader literature.

Discussion of the role of Frazzled in chemical synaptogenesis is missing, along with relevant references including:

Horn et al. (2013) DCC Expression by Neurons Regulates Synaptic Plasticity in the Adult Brain, Cell Reports Tu et al. (2015) C. elegans Punctin Clusters GABAA Receptors via Neuroligin Binding and UNC-40/DCC Recruitment, Neuron Zhou et al. (2020) The netrin receptor UNC-40/DCC assembles a postsynaptic scaffold and sets the synaptic content of GABAA receptors, Nature comm. • We have added a short section on C. elegans as suggested in Lines 575-587. The major distinction between our work and the worm referenced below is that we have very little access to the function of the chemical synapse in the GFS since it has almost no physiological function that we can test.

Specific points below:

Line 13: 'Frazzled fails to rescue the synaptic function' - the 'the' should not be there. • Agreed, this has been removed from the text.

Line 29-30: 'The present work is the first to show how a guidance molecule regulates synaptogenesis through transcriptional regulation of synaptic components' - no it is not. A reviewer brought this up in the last round of review - this needs to be corrected • Agreed, it was a mistake that this got through. It has been corrected in Lines 17-18.

Line 39: A past reviewer made the point that you should credit Hedgecock and colleagues for discovering netrin, but you have the wrong reference. Should be Hedgecock, Culotti and Hall (1990) • The correct reference has been included in Lines 23-24.

Lines 53-54: 'This synapse has been studied since the early 1980s, and the invertebrate gap junctions (GJs) were discovered and characterized there (Blagburn et al., 1999; Phelan et 55 al., 2008; Thomas &Wyman, 1983).' - this writing is unclear - it may be read as if you are suggesting invertebrate GJs were originally discovered at the GF-TTMn synapse - which of course is not the case. • The text has been rearranged and rewritten for clarity, starting from Line 31 of the new version.

Line 57: 'mediated by the innexin gene for gap junctions' - awkward phrasing that is vague and imprecise • This has been rewritten for clarity and precision in Line 40 as: "mediated by innexins produced from the gene shaking-B" Lines 59-60: 'Shaking-B gap junctions are composed of four protein subunits in the presynaptic and four in the postsynaptic membranes (Oshima, 2020; Phelan et al., 2008)' - this is incorrect. Each innexin has four transmembrane helices, but each hemichannel is composed of six subunits. Also the Oshima opinion paper is out of place - it makes no mention of shaking-B. • The Oshima paper is now removed. The revised writing is now in Lines 44-46.

Line 79-80: 'Frazzled may be regulating innexin GJs in the GFs at the transcriptional level and we dissected the Frazzled/DCC receptor' - awkward and redundant use of 'innexin GJs', and the ending does not make sense. • Lines 61-63 were rewritten for clarity, and we removed the mention of transcription.

Line 84: 'We demonstrate that Frazzled's ICD is necessary and sufficient to produce the GF-specific gap junctions shaking-B(neural+16) by quantifying...' - it doesn't quite make sense. Are you saying shaking-B(neural+16) GJs are specific to GFs? Did you mean to write 'gap junctions formed with shaking-B'? Even GF-specific gap junctions doesn't make sense, because the GJs are formed between GFs and TTMns. • The idea we tried to convey with this is that indeed shaking-B(neural+16) forms the only GJs present in the GFs, not that they are only found in the GFs and nowhere else in the fly. They are the only GJs present in the GFs as shown by Allen et. al., 2008: "It transpires that shak-B(neural + 16), rather than shak-B(neural), is expressed in the GFs https://www.sciencedirect.com/science/article/pii/S1084952105001229#bib37. shak-B(lethal) appears to be expressed in the TTMns https://www.sciencedirect.com/science/article/pii/S1084952105001229#bib25 suggesting that the GF-TTMn synapses are molecularly asymmetric." We rewrote this section (Lines 65-67) for clarity and included the shaking-B(neural+16) GJ specificity information in Lines 46-48.

Lines 129-131: 'Approximately half of the GF axons grow aberrantly in the brain and never reach the ventral nerve cord. The remaining...' - in the mutants or in wild-type? • Half of the mutant GF axons. This is rewritten for clarity in Lines 109-110.

Line 154-155: 'Consequently, each principal component was then compared between WT and Bilateral individuals using t-tests.' - were there corrections for multiple comparisons? • Yes, we included the performed MANOVA tests of the PCA scores, alongside Bonferroni, Holm and Benjamini-Hochberg tests in lines 137-139.

Line 162: 'the head case was also opened to reveal the brain' - expose the brain? • Corrected in Line 144.

Lines 166-167: Could the authors state in the text where the anti-shaking-B antibody binds and how it relates to the different isoforms • Lines 150-153 now detail the consensus sequence shared between all three isoforms that the anti-shaking-B antibody binds to.

Lines 178-179: 'Samples were magnified using a 60x oil immersion objective scanned at a pixel resolution of 1024 x 1024, and z-step sizes were 0.125μm.' - it is odd (and not useful to readers) to report the pixel dimensions, especially when the z-step sizes are in µm. • Corrected in Lines 164-654.

Lines 180-181: 'contrast and brightness were adjusted to increase image quality' - the image quality remains the same - you are just stretching the intensities to change how it is displayed • This is now rewritten for clarity in Line 168.

Lines 187-189: 'We applied a gap junction antibody mask to assess the volume in the GF terminals taken up by the gap junction antibody channel mask to show only the GF terminal content, excluding the antibody label outside the terminal.' - the wording is unclear. Also please describe how the mask was generated • Lines 169-177 were rewritten to include the mask generation details and for clarification.

Lines 205-206: please give a short description contained within the methods section of how you ablated • This is now described starting at Line 191.

Lines 211-214: describe how you detected neurobiotin. Also where you purchased it • This is now described in Lines 200-208.

Line 286: 'using various UAS-Frazzled constructs' - another example of vagueness • Corrected to remove vagueness (Lines 275-278).

Line 351: 'Bilateral terminals appeared in most genotypes in these experiments' - excluding wild-type? • Correct. Lines 340-341 now specify: "Bilateral terminals appeared in all mutant genotypes examined and were not observed in wild-type specimens (Figure 1B, 1H)." Lines 352-353: 'When we separate the terminals found in Frazzled LOF specimens into two groups (wild-type-appearing and bilateral), they are functionally indistinguishable' - the description of the PCA comes later in this section, leaving the reader confused at this point because this statement appears unjustified. • Lines 344-355 were rewritten to bring up the PCA description sooner and the paragraph is now much more cohesive.

Line 395: 'Full-length UAS-Frazzled rescues the Giant Fiber electrical synapse' - it partially rescues • Corrected (Line 388).

Lines 415-416: 'This rescue of synaptic structure and function strongly supports our hypothesis that UAS-Frazzled regulates synaptogenesis in the GFs.' - this is presented as a novel hypothesis but is already well established by the work of the authors and others. Also, why UAS-Frazzled? • We agree that Lines 407-409 should say "...that Frazzled regulates synaptogenesis..."The intention behind this was to convey how the UAS-Frazzled experiment results were successfully replicated from Orr et al 2014 and continue to agree with our hypothesis up to this point in the writing; we did not intend to convey novelty. It has been rewritten as: "This rescue of synaptic structure and function agrees with prior findings (Orr et al., 2014) and strongly supports our hypothesis that Frazzled regulates synaptogenesis in the GFs.

Lines 474-475: 'When we analyzed the GF terminals of LOF flies driving UAS-HA-FraE1354A, we found that this construct rescued neither axon guidance nor synaptogenesis' - did the authors stain with anti-HA to verify the protein is expressed? • Yes, we have included confocal images showing anti-HA staining with antibody to show this (Supplemental Figure 1).

Lines 490-491: 'We also tested flies with one copy of the construct in a wild-type background and found no significant difference between them' - where is the data? • We have included the data as a significance test in Lines 480-485.

Lines 497-499: 'As the amount of gap junction was reduced in the experimental animals, the latency and rate of response decrement both increased (Figure 4A, 4C and 4D). Since gap junctions are not typically use-dependent, the increased rate of response decrement (Figure 4C) was an enigma.' - the authors use the phrase 'response decrement' which I think refers to less reliability/greater latency. I find the phrasing imprecise. Agree GJs are not typically use-dependent - but include citation. Calling it an 'enigma' is unusual for the results section of a scientific paper, but if the authors want that then fine. • In Lines 490-491, we removed the use of enigma and reorganized this section for clarity and removed the use of response decrement: "As the amount of gap junction was reduced in the experimental animals, stimulus response failures and successful response latency both increased (Figure 4A, 4C and 4D)." Lines 504-505: 'One prominent feature of function is the dramatic response decrement induced in multiple genotypes exhibiting different levels of gap junction in the terminals' - I'm not sure what you mean by 'one prominent feature of function' - it comes across as nonsensical • This has been removed entirely from the text.

Lines 542-543: 'The results confirm our hypothesis that Frazzled/DCC regulates synaptogenesis in the Giant Fibers by regulating gap junctions' - this is presented as a novel hypothesis, but is already well established by the work of the authors and others • Lines 529-532 were adjusted to rephrase the opening of the discussion: "The results reinforce prior findings and provide novel mechanistic insight into how Frazzled regulates synaptogenesis in the Giant Fibers." Lines 546-548: 'By combining electrophysiological data and confocal imaging, we show how Frazzled regulates the assembly of electrical synapses.' - this is a misleading overstatement. The authors show that the frazzled intracellular domain is able to rescue, to some degree, the loss-of-function phenotypes, and a construct with a point mutation in the transcriptional activation domain is unable to rescue, suggesting something to do with transcription. • Based on the results, we argue that the Frazzled intracellular domain (ICD) rescues the loss-of-function phenotypes such that there is no significant difference in physiology or gap junction staining between the mutants expressing UAS-FraICD and wild-type flies. The partial rescue comes from UAS-Frazzled. We do not see how this is an overstatement of the ICD's results. Nevertheless, we have adjusted the phrasing in Lines 534-536 to the following: "By combining electrophysiological data and confocal imaging, we show strong evidence of a role for Frazzled in regulating electrical synapses in the GFs." Line 555: 'antibodies to gap junctions' - be specific • Lines 542-545 now read: "Our shaking-B antibody results show that shaking-B gap junctions make up 9.04% of GF terminal volume in control siblings and 5.14% of GF terminal volume in LOF mutants (Figure 4B)." Lines 730-731: 'Model Author. (2025). XXXXXXXXXXXXXXXXXXXXXXXXXXXXXXXX [Computer software]. https://nam02.safelinks.protection.outlook.com/?url• This is now properly referenced.

Line 812: 'Figure 1. Axon guidance phenotypes and resulting physiology' - 'physiology' is used throughout the paper and is vague • Corrected to "Axon guidance phenotypes and construct schematics" in Line 659.

Lines 826-827: 'UAS-FraE1354A contains an HA tag that does not influence synaptic function (not shown).' - show it • Edited to show the location of the HA tag in the construct (mentioned in Lines 673-674).

Line 841: 'Compressed z-stack projection image' - what kind of z-stack? Maximum intensity? Average intensity? Other? • Added Maximum intensity in the methods (Line 167).

Lines 842-843: 'shaking-B(neural+16) gap junctions labeled with shaking-B antibody' - does the antibody bind specifically to neural+16 or to all shaking-B innexins? • Edited to show that the antibody binds to all shaking-B innexins.

Figure 1 A-G: it would be much easier for readers if the genotype was labelled on the panels Figure 1H-I: The orders of the genotypes are mixed up between H and I, making it harder to read. Also in panel I showing the different proteins expressed by the different constructs, the UAS is not particularly relevant • Edited panel I to follow order of panel H, and removed UAS from construct names.

Figure 2C and Figure 3 right panels: scale for recordings • Scale has been added.

Figure 3: A cartoon of the Frazzled protein variant would be a useful addition • We have adjusted the legend for Figure 3 to now reference Figure 1I every time a Frazzled construct is presented: "A representation of this construct is found in Figure 1I." Figure 4C: axis labels? • These have been added back to the panel.
