# Supplementary figures and images for: Frazzled/DCC Regulates Gap Junction Formation at a Drosophila Giant Synapse
Source: eNeuro. 2025 Oct 23;12(10):ENEURO.0202-25.2025. doi: 10.1523/ENEURO.0202-25.2025 (PMC12570126; doi:10.1523/ENEURO.0202-25.2025)

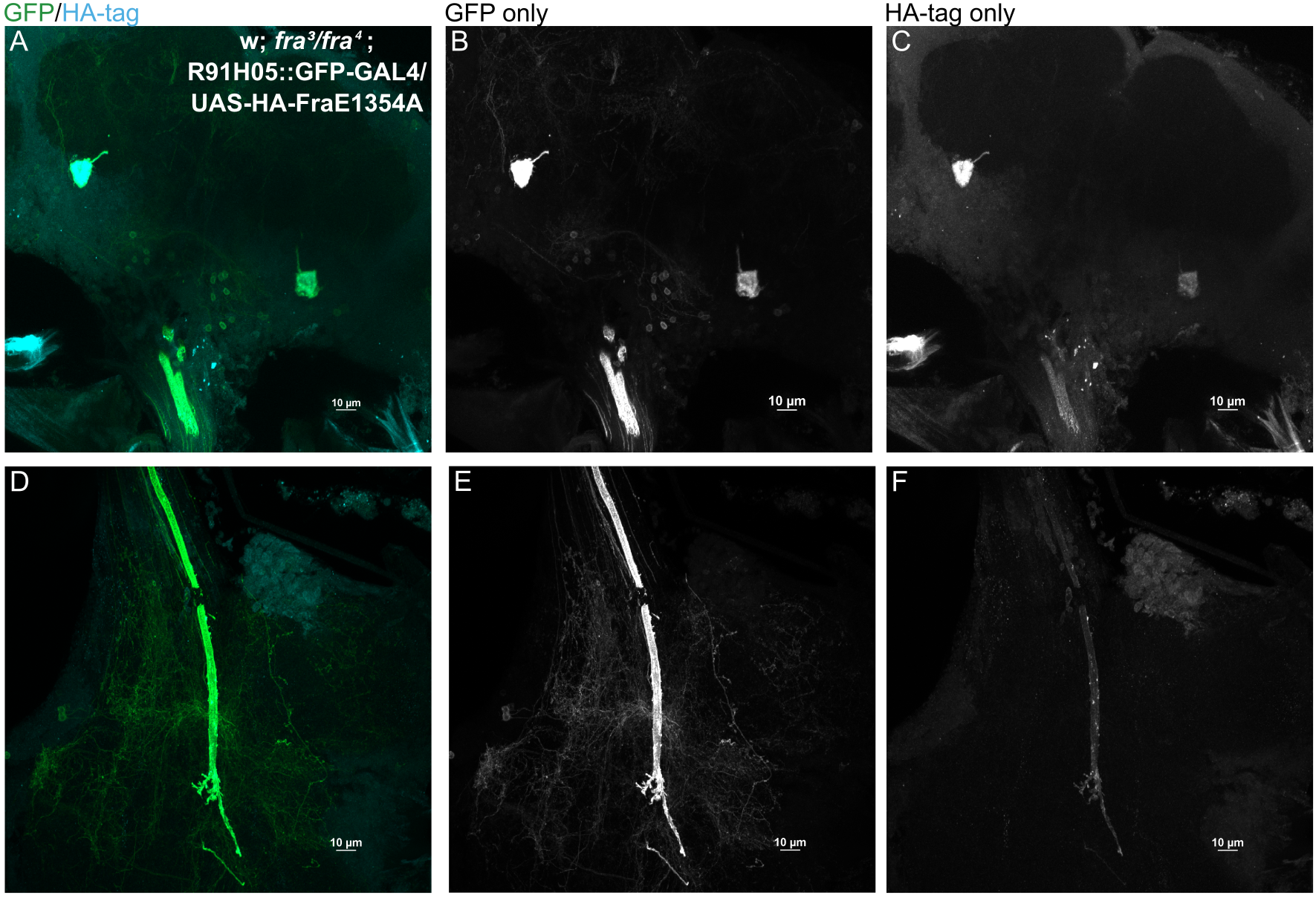

Supplement: Figure 1-1 — HA- tag immunolabeling in a frazzled LOF mutant driving expression of UAS-HA-FraE1354A in the Giant Fibers. Figures show expression of UAS-HA-FraE1354A genetic construct. All panels are the same genotype. The top and bottom rows are different samples. A) Sample showing expression of GFP and anti-HA in the brain of Drosophila, with GFP in green and anti-HA in cyan. B) GFP expression channel. C) Anti-HA expression channel. D) Different sample showing expression of GFP and anti-HA in a single GF. E) GFP expression channel. F) Anti-HA expression channel. Download Figure 1-1, TIF file. [file eneuro-12-ENEURO.0202-25.2025-s002.tif]
